# *MorphoSim*: an efficient and scalable phase-field framework for accurately simulating multicellular morphologies

**DOI:** 10.1038/s41540-023-00265-w

**Published:** 2023-02-17

**Authors:** Xiangyu Kuang, Guoye Guan, Chao Tang, Lei Zhang

**Affiliations:** 1 Center for Quantitative Biology, Peking University, Beijing, 100871 China; 2 Peking-Tsinghua Center for Life Sciences, Peking University, Beijing, 100871 China; 3 School of Physics, Peking University, Beijing, 100871 China; 4 Beijing International Center for Mathematical Research, Peking University, Beijing, 100871 China; 5 Center for Machine Learning Research, Peking University, Beijing, 100871 China

**Keywords:** Synthetic biology, Computational science

## Abstract

The phase field model can accurately simulate the evolution of microstructures with complex morphologies, and it has been widely used for cell modeling in the last two decades. However, compared to other cellular models such as the coarse-grained model and the vertex model, its high computational cost caused by three-dimensional spatial discretization hampered its application and scalability, especially for multicellular organisms. Recently, we built a phase field model coupled with in vivo imaging data to accurately reconstruct the embryonic morphogenesis of *Caenorhabditis elegans* from 1- to 8-cell stages. In this work, we propose an improved phase field model by using the stabilized numerical scheme and modified volume constriction. Then we present a scalable phase-field framework, *MorphoSim*, which is 100 times more efficient than the previous one and can simulate over 100 mechanically interacting cells. Finally, we demonstrate how *MorphoSim* can be successfully applied to reproduce the assembly, self-repairing, and dissociation of a synthetic artificial multicellular system - the *synNotch* system.

## Introduction

The multicellular systems formed by physically interacting cells are widespread in animals, plants, and microorganisms like fungi and choanoflagellates^1–4^. They usually consist of multiple cell types and characteristic spatial organizations, ranging in scales from tissue, and organ, to an individual, and are often involved with embryogenesis and organogenesis^5,6^. For example, the mouse valley-like small intestine, which contains ~250 cells with six cell types in specific proportions, plays a role in nutrient uptake and has strong architectural robustness and regenerative capacity^7–9^. The mechanics of interacting cell aggregates, as well as their morphological and morphogenetic effects, has attracted increasing attention in the fields of cell biology, developmental biology, cancer biology, etc.^10–12^. Apart from the natural ones, many engineered systems have been constructed in vitro artificially, such as the organoid and embryoid used for developmental biology research and high-throughput drug test^13–15^. Furthermore, synthetic biologists are trying to program the cellular interaction de novo to build customized multicellular living machines, robots, or patterns, using bottom-up or top-down engineering approaches. The *synNotch* and *Xenobot* systems are two cutting-edge representatives, which are constructed for designed functions using hundreds of animal cells and have broad application areas like medical treatment and synthetic development^16–19^. An urgent need for a computational tool that can efficiently and accurately simulate multicellular morphological behaviors is emerging^20,21^.

Many physical models have been utilized to study the morphological dynamics of multicellular systems, including cellular automaton^22^, cellular Potts model^23^, coarse-grained model^24^, Voronoi tessellation model^25^, vertex model^26^, multi-particle model^27^, phase field model^28–30^, etc. These models have provided much mechanistic knowledge and many insights for understanding biological processes. However, simulation results of the same system may vary from model to model—when a precise description of the real system is critical the accuracy of the model output becomes an important issue^31^. Besides, most of the existing models often lack detailed validation by quantitative comparison with experiments, especially at three-dimensional (3D) and single-cell levels. Among the existing multicellular models, the phase field model, which uses a continuous diffusion field to describe a cell, is emerging quickly these years for it can comprehensively characterize cell shapes and interface-based cell–cell interactions^32–38^ and cell-substrate interactions^39,40^ without explicitly tracking the cell interface; besides, it can incorporate intracellular fluid dynamics^41^, biochemical regulatory mechanisms such as the reaction-diffusion of interacting molecules^42^, and the actin flow that mediates cell motility^40^.

**Fig. 1:**
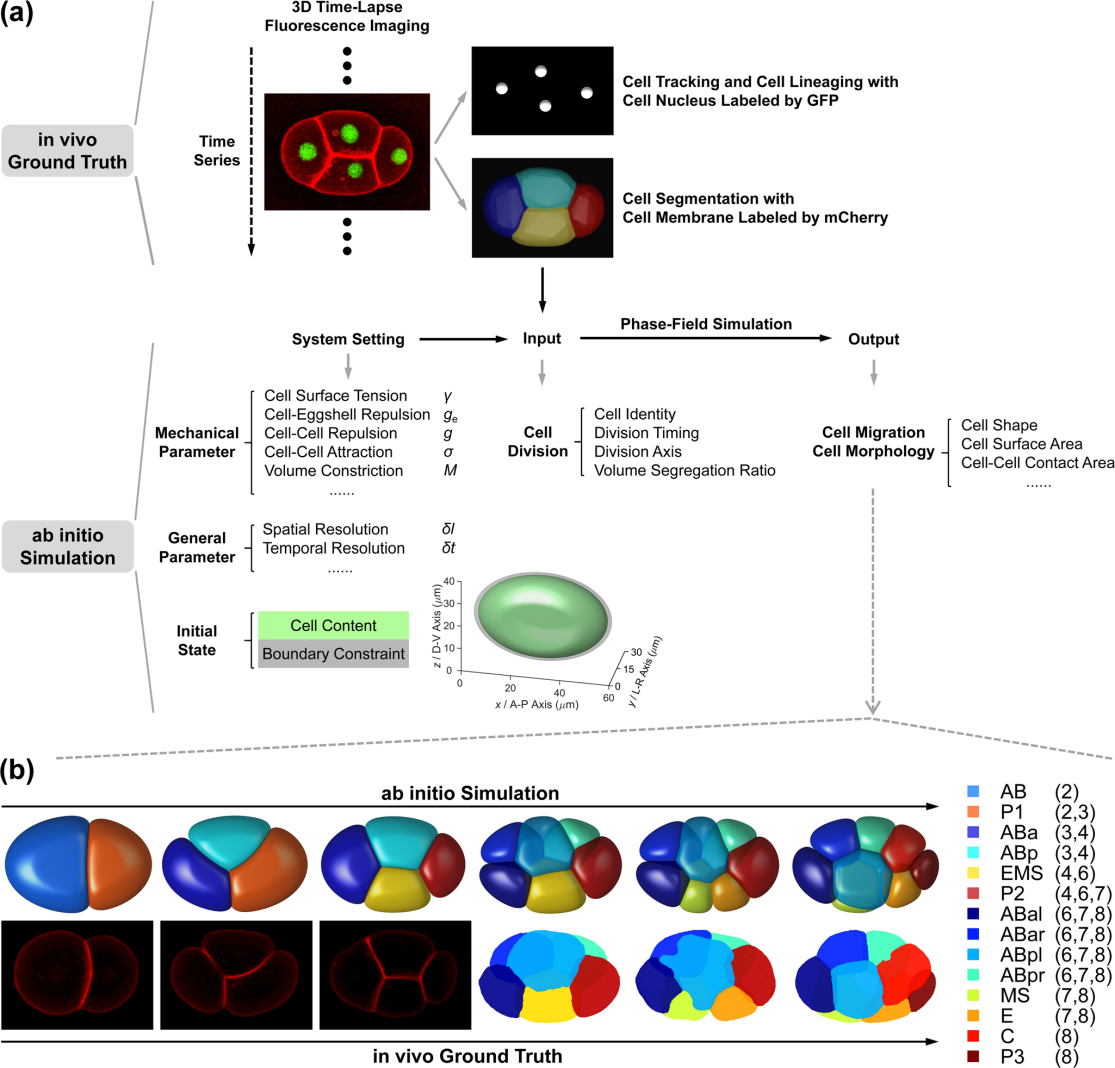
The phase-field framework for *C. elegans* early embryogenesis. **a** Flow chart. **b** Comparison between ab initio simulation and in vivo ground truth of *C. elegans* embryonic morphologies from 2- to 8-cell stages. The embryonic structures at 2-, 3-, and 4-cell stages are two-dimensional and shown by fluorescence images; the ones at 6-, 7-, and 8-cell stages are three-dimensional and shown by segmented images. The cells’ identities and corresponding colors and existing stages (represented by the total cell number of the stage) are denoted on the right. All the subfigures, except the schematics for the initial state setting in (a), are adapted from ref. ^43^ with granted permission.

Given that the roundworm *Caenorhabditis elegans* embryo has stereotypic cell arrangement patterns among individuals and lots of imaging data about its embryogenesis has been collected, the phase field model has been introduced into this system with quantitative comparisons between in silico and in vivo morphologies being carried out^43–46^. On the one hand, Seirin-Lee et al. (2022) devised a phase field model that considers cell surface tension, cell–eggshell and cell–cell repulsion, cell–cell attraction, and cell volume constriction; they successfully reproduced the T-reverse-shaped pattern observed in RNAi-treated 4-cell embryos with a slim eggshell and eliminated cell adhesion, which however failed to be reproduced by a coarse-grained model as reported before^46,47^. On the other hand, Kuang et al. (2022) constructed a 3D phase field model with the cell morphology data collected by fluorescence imaging and membrane segmentation (Fig. 1a)^43,48^; the model can accurately regenerate the in vivo cell morphologies and the conserved cell–cell contact map and infer the underlying biophysical properties (e.g., intercellular adhesion) from 1- to 8-cell stages (Fig. 1b). Compared to another two mechanical models established in *C. elegans* embryogenesis research, i.e., the multi-particle model (that describes cell shape with many interacting particles and many parameters^27,49^) and the coarse-grained model (that describes a cell as a single particle and neglects the cell shape)^47,50–53^, the phase field model can both simulate the cell shape and shape-associated interaction and has a considerable number of biophysically-significant parameters. Despite its outstanding performance, the model is still limited by the high computational cost caused by spatial discretization and increasing cell numbers, especially when the cell number reaches dozens and hundreds.

In this work, we propose an efficient and scalable phase-field framework that can accurately simulate multicellular morphologies. We first develop a stabilized numerical scheme that allows for large-time steps. Next, we enhance the precision of cell volume control to avoid “cell disappearance”. Finally, a phase-field framework, *MorphoSim*, is established along with Matlab-based software. By testing on the simulations of *C. elegans* embryogenesis, it can achieve a computational efficiency of more than 100 times the previous one and is capable of computing over 100 cells. As an application, the *MorphoSim* framework can successfully reproduce the assembly, self-repairing, and dissociation of the *synNotch* system reported in ref. ^16^.

## Results

### Review of the phase field model

The original phase field model adopted by this study considered the surface tension ${{{\boldsymbol{F}}}}_{{{{\mathrm{ten}}}}}$ and volume constriction ${{{\boldsymbol{F}}}}_{{{{\mathrm{vol}}}}}$ imposed on a cell, and the repulsion ${{{\boldsymbol{F}}}}_{{{{\mathrm{rep}}}}}$ and attraction ${{{\boldsymbol{F}}}}_{{{{\mathrm{atr}}}}}$ between cells^29,30,43,54^. The spatial constraint from the eggshell is also repulsive to a cell toward the embryo’s center and thus is included in the term ${{{\boldsymbol{F}}}}_{{{{\mathrm{rep}}}}}$; the eggshell boundary is set as a truncated ellipsoid fitted with experimental data (Fig. 1a and Supplementary Note 1)^43,48^. For a system composed of *N* cells, the *i*-th cell with a prescribed volume $V_i\left( t \right)$ is represented by a phase field $\phi _i\left( {{{{\boldsymbol{r}}}},t} \right)$ and assumed to deform and migrate in an overdamped domain Ω, following the governing equations:

1 $${{{\boldsymbol{F}}}}_{{{{\mathrm{ten}}}}} = - \gamma \left( {{{\Delta }}\phi _i - cW^\prime \left( {\phi _i} \right)} \right)\frac{{\nabla \phi _i}}{{\left| {\nabla \phi _i} \right|^2}},$$

2 $${{{\boldsymbol{F}}}}_{{{{\mathrm{vol}}}}} = M\left( {{\int}_\Omega {\phi _i} {{{\mathrm{d}}}}{{{\boldsymbol{r}}}} - V_i\left( t \right)} \right){{{\hat{\boldsymbol n}}}},$$

3 $${{{\boldsymbol{F}}}}_{{{{\mathrm{rep}}}}} = \left( {g_{{{\mathrm{e}}}}\phi _i\phi _{{{\mathrm{e}}}}^2 + g\phi _i\mathop {\sum }\limits_{j \ne i}^N \phi _j^2} \right)\frac{{\nabla \phi _i}}{{\left| {\nabla \phi _i} \right|^2}},$$

4 $${{{\boldsymbol{F}}}}_{{{{\mathrm{atr}}}}} = \mathop {\sum }\limits_{j \ne i}^N \sigma _{i,j}\nabla \phi _j,$$

5 $$\frac{{\partial \phi _i}}{{\partial t}} = - \frac{1}{\tau }\left( {{{{\boldsymbol{F}}}}_{{{{\mathrm{ten}}}}} + {{{\boldsymbol{F}}}}_{{{{\mathrm{vol}}}}} + {{{\boldsymbol{F}}}}_{{{{\mathrm{rep}}}}} + {{{\boldsymbol{F}}}}_{{{{\mathrm{atr}}}}}} \right) \cdot \nabla \phi _i,$$

Here Δ and ∇ are Laplacian and gradient operators respectively; *γ* denotes the cell surface tension that drives the cell shape to be spherical; *c* controls the cell boundary thickness; $W\left( \phi \right) = \phi ^2\left( {\phi - 1} \right)^2$ separates two phases at $\phi = 0$ and $\phi = 1$, corresponding to the exterior and interior of a cell; on the contrary, $\phi _{{{\mathrm{e}}}}$, the phase field of the eggshell, describes its outer space with 1 and its inner space with 0, consequently constraining the phase fields of cells and keeping the multicellular system evolving inside the eggshell; $g_{{{\mathrm{e}}}}$ and *g* represent the cell–eggshell and cell–cell repulsions respectively; $\sigma _{i,j}$ represents the attraction strength between the *i*-th and *j*-th cells; $V_i\left( t \right)$ is the target volume of the *i*-th cell at the specific time point *t* and the designated volume can be obtained from experimental measurement or set automatically; *M* is the volume constriction strength; ${{{\hat{\boldsymbol n}}}}$ is the unit normal vector at the cell surface and orients inward; *τ* is the ambient viscosity.

Division of the *i*-th cell is simplified as an instant splitting of the phase field denoted as $\phi _i^ \ast$ into two new regions, according to a certain splitting plane. The plane is normal to the cell division axis ***n*** and is located in ${{{\boldsymbol{n}}}} \cdot \left( {{{{\boldsymbol{r}}}} - {{{\boldsymbol{r}}}}_{{{\mathrm{c}}}}} \right) - b = 0$, where ${{{\boldsymbol{r}}}}_{{{\mathrm{c}}}} = \frac{{{\int}_\Omega {{{{\boldsymbol{r}}}}\phi _i^ \ast {{{\mathrm{d}}}}{{{\boldsymbol{r}}}}} }}{{{\int}_\Omega {\phi _i^ \ast {{{\mathrm{d}}}}{{{\boldsymbol{r}}}}} }}$ is the center of $\phi_i^\ast$ and *b* is uniquely determined by setting a designated volume ratio to the two separate regions. Subsequently, the division of the phase field of the *i*-th cell is implemented as follows^55^:

6 $$\phi _{N + 1} = \phi _i^ \ast \left( {\frac{{{{{\mathrm{tanh}}}}\frac{{{{{\boldsymbol{n}}}} \cdot \left( {{{{\boldsymbol{r}}}} - {{{\boldsymbol{r}}}}_{{{\mathrm{c}}}}} \right) - b}}{{\it{\epsilon }}} + 1}}{2}} \right),$$

7 $$\phi _i = \phi _i^ \ast \left( {\frac{{{{{\mathrm{tanh}}}}\frac{{b - {{{\boldsymbol{n}}}} \cdot \left( {{{{\boldsymbol{r}}}} - {{{\boldsymbol{r}}}}_{{{\mathrm{c}}}}} \right)}}{{\it{\epsilon }}} + 1}}{2}} \right).$$

where $\phi _{N + 1}$ and $\phi _i$ are the initial state of the two phase fields generated by cell division; *ϵ* represents the width of the splitting interface.

During *C. elegans* embryogenesis, every cell has unique and identifiable developmental behavior and is systematically named based on its cell type, lineal origin, and spatial location^44^. Remarkably, the confocal 3D time-lapse fluorescence imaging on the cell nucleus and cell membrane has enabled cell-resolved monitoring for *C. elegans* embryogenesis at ~1.5 intervals (Fig. 1(a))^48^. On the one hand, the cell tracking and cell lineaging based on the GFP-labeled cell nucleus provides information on cell identity, cell division timing and order, and cell division axis; on the other hand, the cell segmentation based on the mCherry-labeled cell membrane provides the volume of each cell and the volume segregation ratio in each cell division. Besides, fluorescence labeling on adhesive protein HMR-1 has shown that it’s rarely accumulated in the newly-formed membrane between sister cells and in some specific cell–cell contacts, including EMS-P2 at the 4-cell stage and ABpl-E at the 8-cell stage^47,56^; for the simplicity, the cell–cell attraction in the phase field model is binarized into relatively weak ($\sigma _{{{\mathrm{W}}}}$) for the abovementioned contacts and strong ($\sigma _{{{\mathrm{S}}}}$) for the others^43^. With the input of cell division order and axis and volume segregation ratio measured experimentally (Supplementary Table 1), the previous phase-field framework based on Eqs. (1–7) successfully reproduced the typical embryonic morphologies seen in the experiment up to the 8-cell stage (Fig. 1b)^43^. The conserved cell–cell contact map observed in vivo was fully reproduced and the regulatory programs on cell–cell adhesion were reversely inferred, including the relatively weak adhesion in EMS-P2 and ABpl-E contacts reported before^47,56^. Those results have validated the applicability of the mathematical forms of the governing equations. The simulation pipeline about how the cell–cell adhesion is assigned and inferred and how the cell division timing is set for each stage is summarized in Supplementary Note 1. To distinguish the time scales with different meanings, hereafter the time in the experiment and computer are referred to as “in vivo time” and “in silico time” (in silico time = step number × step size = $n_t\delta t$) respectively, while the time cost for simulation is termed “computing time” and is one of the optimization targets in this work.

### Stabilized numerical scheme

The high computational cost of phase-field simulation is largely attributed to its 3D spatial discretization and cell number increase. One direct attempt to reduce the computational cost is to minimize the spatial and temporal resolutions while guaranteeing the results are consistent with the previous biological findings^43^. When adopting the parameter assignments from the original framework^43^ (Supplementary Table 2) and inputting the cell division order and axis and volume segregation ratio from in vivo *C. elegans* embryos^48^ (Supplementary Table 1), the requirements that judge if the framework works precisely enough from the 1- to 8-cell stages mainly include three parts: (1) the cell–cell contact maps are the same as the ones conserved between individual embryos (Supplementary Fig. 1a–d); (2) the previously reported cell–cell adhesion programs (i.e., relatively weak adhesion between EMS and P2 cells at the 4-cell stage and between ABpl and E cells at 8-cell stage) can be inferred by morphological comparison to experiment and parameter scanning (Fig. 1b and Supplementary Figs. 2a, b, 3a–c)^47,56^; (3) the embryonic morphologies resemble the ones in vivo (Fig. 1b and Supplementary Fig. 3a, b). The details of the simulation procedure and quantitative criteria are introduced in Supplementary Note 1. Given the original spatial grid size $\delta l = 0.25$ ${{{\mathrm{\mu m}}}}$ and time step size $\delta t = 0.10$ (corresponding to 0.0036 s in vivo) from ref. ^43^, we scan *δl* from 0.25 to 2. 00 μm and *δt* from 0.1 to 0.4 by repeating the simulation procedure from 1- to 8-cell stages as established in Supplementary Note 1 and Supplementary Fig. 4a. In each simulation, the root-mean-square velocity of all cells’ mass centers (i.e., $\bar v$) is calculated at each time step and used for triggering the cell division(s) in an experimentally-observed order (Table 1). For 1-, 2-, 3-, and 4-cell stages, the next round of cell division(s) takes place when the system reaches its steady state (defined by $\bar v \,<\, 1 \times 10^{ - 4}$); for 6- and 7-cell stages, the system is allowed to evolve to its first quasi-steady state (defined by ${\frac{{{{{\mathrm{d}}}}\bar v}}{{{{{\mathrm{d}}}}t}}} \big|_{t = t_{{{\mathrm{q}}}}} = 0, {\frac{{{{{\mathrm{d}}}}^2\bar v}}{{{{{\mathrm{d}}}}t^2}}}\big|_{t = t_{{{\mathrm{q}}}}}\, >\, 0$) and then cell division(s) takes place; finally, the 8-cell stage lasts for a constant in silico time = 15,000 (Supplementary Note 1)^43^. As a result, we find that the three requirements can be satisfied by a coarser spatial grid size $\delta l = 0.50$ ${{{\mathrm{\mu m}}}}$ (grid nodes in *x*, *y*, and *z* axes: 120 × 60 × 80) and time step size $\delta t = 0.30$ (Fig. 4b, c). However, such improvement is very limited.

**Table 1:** Computing time in GPU and CPU using the previous and current frameworks.

Cell Stage	Computing time			
	Previous framework^43^		Current framework (*MorphoSim*)	
	GPU^a^	CPU^b^	GPU^a^	CPU^b^
6-Cell Stage	35.3 min	1428.0 min	0.3 min	4.1 min
7-Cell Stage	30.9 min	1116.1 min	0.1 min	2.0 min
8-Cell Stage	229.8 min	7767.8 min	2.6 min	39.5 min
25-Cell Stage	854.3 min	26067.4 min	17.2 min	186.6 min
50-Cell Stage	—^c^	67629.4 min	29.8 min	356.3 min
100-Cell Stage	—^c^	—^c^	93.8 min	706.1 min

^a^GPU, NVIDIA Tesla P100.

^b^CPU, Inter Xeno E5-2680 v3.

^c^“—” represents “out of memory”.

The stability restriction on the time step size impedes the further acceleration of computation. In order to allow a much larger time step size than the explicit schemes, we first adopt a first-order semi-implicit scheme^57^:

8 $$\frac{\tau }{{\delta t}}\left( {\phi _i^{n + 1} - \phi _i^n} \right) = \gamma {{\Delta }}\phi _i^{n + 1} + F_i^n,$$

where *n* denotes the *n*-th time step and the Fourier spectral method is used for the spatial discretization throughout this work. The linear term $\gamma {{\Delta }}\phi _i^{n + 1}$ was treated implicitly while the nonlinear term $F_i^n$ is treated explicitly and expressed by:

9 $$\begin{array}{ll}F_i^n = - \gamma c\left( {4\left( {\phi _i^n} \right)^3 - 6\left( {\phi _i^n} \right)^2 + 2\phi _i^n} \right) - g_{{{\mathrm{e}}}}\phi _i^n\phi _{{{\mathrm{e}}}}^2 - g\phi _i^n\mathop {\sum }\limits_{j \ne i}^N \left( {\phi _j^n} \right)^2 \\\quad\qquad-\, \nabla \phi _i^n \cdot \mathop {\sum }\limits_{j \ne i}^N \sigma _{i,j}\nabla \phi _j^n + M\left( {V_i\left( t \right) - \mathop {\int}\limits_\Omega {\phi _i^n} {{{\mathrm{d}}}}{{{\boldsymbol{r}}}}} \right)\left| {\nabla \phi _i^n} \right|,\end{array}$$

**Fig. 2:**
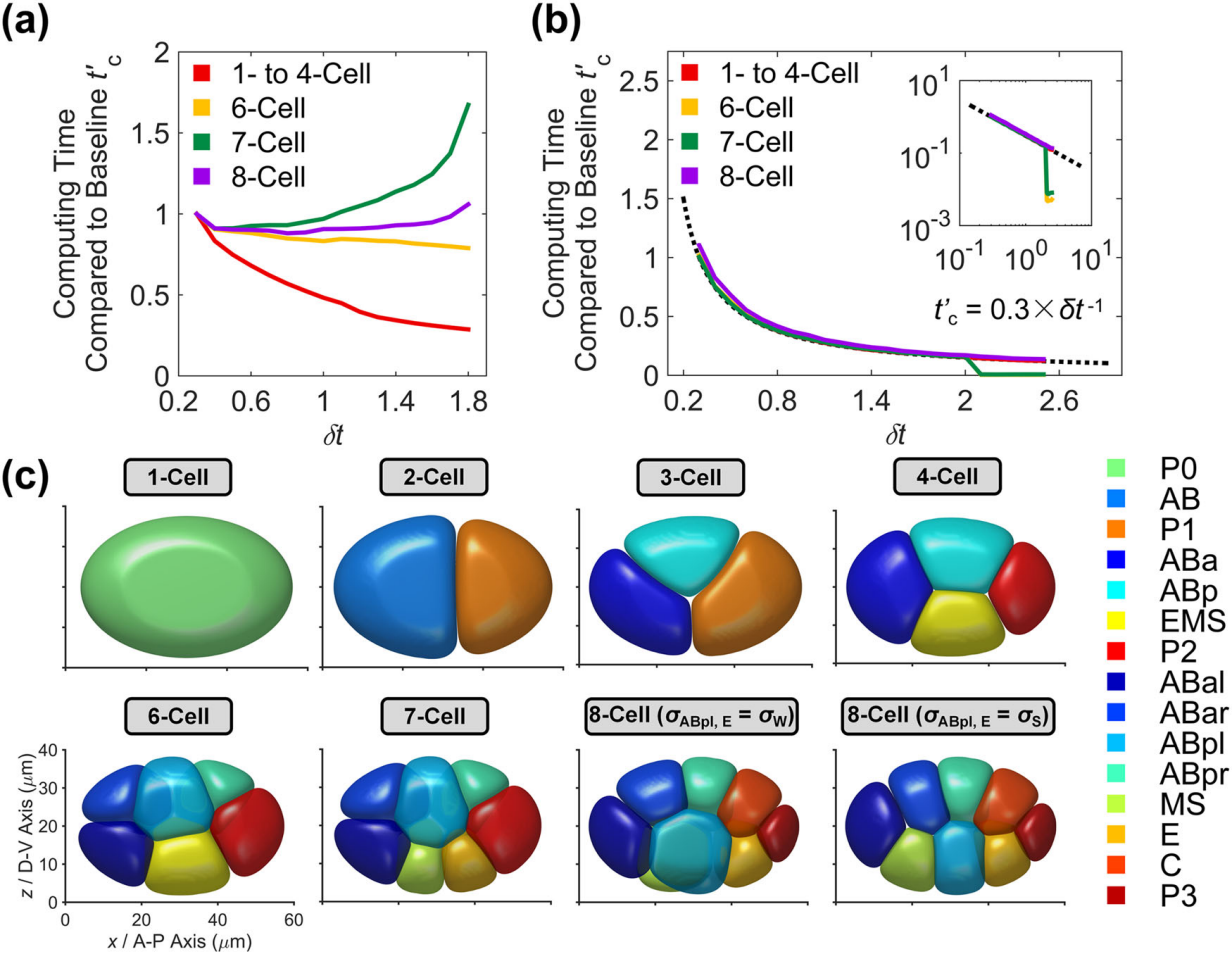
Framework improvement by the addition of stabilization term. **a** Computing time compared to the baseline at different stages in the first-order scheme. **b** Computing time compared to the baseline at different stages in the second-order scheme. In both (**a**) and (**b**), the baseline is set as the computing time when $\delta t = 0.3$ and no stabilization term is added. In **b**, the dashed line is the curve $t^\prime _{{{\mathrm{c}}}} = 0.3 \times \delta t^{ - 1}$ and the inset is the same figure presented in a dual logarithmic coordinate system. **c** Embryonic morphologies from 1- to 8-cell stages in the second-order scheme with $\delta t = 2.0$. The cells’ identities and corresponding colors are denoted on the right; $\sigma _{{{\mathrm{S}}}}$ and $\sigma _{{{\mathrm{W}}}}$ denote the relatively strong (i.e., $\sigma _{{{\mathrm{S}}}} = 0.5$) and weak (i.e., $\sigma _{{{\mathrm{W}}}} = 0$) cell–cell attraction inferred at the 4-cell stage respectively (Supplementary Fig. 2a, b and Supplementary Note 1).

Next, we relax the restriction by the stabilization method. The main idea is to add an artificial stabilization term that has a dissipative effect to balance the instability caused by the explicit treatment of the nonlinear term^58,59^. Here, we first introduce a first-order stabilization term $- S\left( {\phi _i^{n + 1} - \phi _i^n} \right)$ to alleviate the strict constraint on temporal evolution:

10 $$\frac{\tau }{{\delta t}}\left( {\phi _i^{n + 1} - \phi _i^n} \right) = \gamma {{\Delta }}\phi _i^{n + 1} + F_i^n - S\left( {\phi _i^{n + 1} - \phi _i^n} \right),$$

where *S* is a positive coefficient proportional to the dissipative effect. Increasing *S* will make computation stabler but also introduce extra numerical error. Thus, it is necessary to keep a balance between stability and accuracy. Therefore, we optimize *S* by finding its minimal value that can stabilize a simulation with *δt* > 0.3 while preserving enough accuracy, i.e., still reconstructing the morphogenetic dynamics observed experimentally (Fig. 1b). For each *δt* from 0.3 to 2.0 in a step of 0.1, we perform simulation from 1- to 8-cell stages to search for the optimal *S* value in a step of 0.1. Here, the time scale of each simulation is proportionally fitted to the one without stabilization term ($\delta t = 0.3$; baseline), using the duration of 6- and 7-cell stages determined by their first quasi-steady states; then the in silico time for the 8-cell stage is calculated with the linear relationship given the base value set as 15,000. It’s shown that the system bifurcates into another 8-cell topology when *δt* exceeds 1.8 (Supplementary Fig. 5a). Besides, with the increment of *δt*, the computing time compared to the baseline (i.e., $t^\prime _{{{\mathrm{c}}}}$) is not always monotonously decreasing (Fig. 2a and Supplementary Table 3), revealing that the in silico time $n_t\delta t$ for the same stage/process also increases (Supplementary Fig. 5b and Supplementary Table 4). This overdamping-like effect, i.e., the increase of in silico time $n_t\delta t$, was also shown in previous research and may be caused by the numerical error of the stabilization term in the first-order scheme, which then limits the computing time reduction gained from a larger *δt*^60^.

To solve the problem above, we further introduce a second-order semi-implicit scheme with a stabilization term to achieve less numerical error^41,61^:

11 $$\frac{\tau }{{2\delta t}}\left( {3\phi _i^{n + 1} - 4\phi _i^n + \phi _i^{n - 1}} \right) = \gamma {{\Delta }}\phi _i^{n + 1} + 2F_i^n - F_i^{n - 1} - S\left( {\phi _i^{n + 1} - 2\phi _i^n + \phi _i^{n - 1}} \right).$$

We extend the scanning range of *δt* to 0.3–2.5 and adopt the same optimization procedure for finding the optimal *S* value. The second-order scheme takes full advantage of the larger *δt* by avoiding the overdamping-like effect, therefore, substantially reducing the computing time and keeping the simulations with different *δt* values scalable (Fig. 2b and Supplementary Table 3). The perfect scalability, which faithfully follows the relationship $t^\prime _{{{\mathrm{c}}}} = 0.3 \times \delta t^{ - 1}$ with a goodness of fit larger than 0.95 for all the stages, is suddenly broken when *δt* exceeds 2.0, probably due to numerical error accumulation. Thus, we choose the time step size *δt* = 2.0 with *S* = 12 as the optimal condition for further simulation, which can still recapitulate the 1- to 8-cell morphogenesis of *C. elegans* embryo (Fig. 2c). Although the second-order scheme gains an edge over the first-order one in computing time, it’s worth pointing out that the first-order scheme is better at maintaining numerical stability^59^. In other words, the first-order scheme allows larger values assigned to the parameters. Note that the *δt* and *S* values presented here are optimized to minimize the computing time, and one may select other values according to the actual problems or parameter settings.

### A new formation of volume constriction to avoid “cell disappearance”

**Fig. 3:**
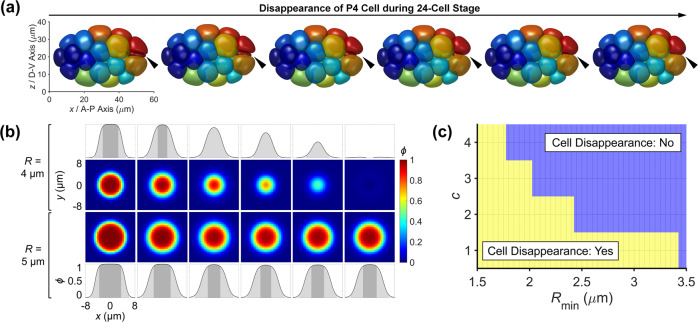
Cell disappearance that happens when the cell size is too small. **a** Gradual disappearance of the P4 cell during the 24-cell stage; the P4 cell is colored dark red and pointed by an arrowhead. **b** Phase field distribution in 1D (*x* axis) and 2D (*xy* plane) of a free cell with a radius of 4 μm (upper two rows) and 5 μm (lower two rows). The single-cell simulation is conducted with open boundaries and illustrated with an in silico interval of 20 from left to right. In the 1D distribution, the boundary of a cell ($0.07 \le \phi\, <\, 0.93$) is painted with light gray while the interior ($\phi \ge 0.93$) and exterior ($\phi \,<\, 0.07$) of a cell are painted with dark gray and white respectively. **c** A scanning on parameter *c* with different cell radii *R*_min_; yellow and blue indicate if the cell disappears or not in a single-cell simulation.

After the improvement of the numerical scheme, we then perform simulations for the later stages of *C. elegans* embryogenesis to find the maximum cell number afforded by the current framework. With the experimentally-measured cell division order and axis and volume segregation ratio inputted (Supplementary Table 1), the simulation proceeds properly until the 24-cell stage, when the whole phase field of the P4 cell, $\phi _{{{{\mathrm{P}}}}4}$, shrinks to zero erroneously (Fig. 3a and Supplementary Movie 1). Hereafter, such a phenomenon caused by numerical error is referred to as “cell disappearance” in this work. In the simulations, cell disappearance is quantitatively defined when a cell’s phase field is globally smaller than 0.5.

When cell disappearance occurs, the volume constriction (Eq. (2)) fails to prevent a cell’s phase field from dropping to the homogeneous state $\phi \equiv 0$. Further simulation on a single cell with different sizes and without any external force reveals that a threshold of cell size exists to determine if cell disappearance happens (Fig. 3b). During the relaxation of a free cell, its interior always shrinks, while the width of the diffusing interface remains nearly constant. If the cell size is below the threshold ($R \le 4$ ${{{\mathrm{\mu m}}}}$ or $V \le 268$ ${{{\mathrm{\mu m}}}}^3$), the interior of the phase field shrinks to disappear and its boundaries gradually overlap, making the phase field eventually converge to $\phi \equiv 0$, namely, cell disappearance (Fig. 3b). As a small cell size appears in all kinds of biological processes in vivo, like blastomere cleavage and cell apoptosis, solving the problem of cell disappearance is vital for simulations of those scenes^62,63^.

The phenomenon of cell disappearance can be avoided in two ways. First, we replace the volume constriction (Eq. (2)) with a new formulation based on the relative error of volume, instead of the absolute error controlled in the previous one:

12 $$F_{{{{\mathrm{vol}}}}} = M^\prime \left( {\frac{{{\int}_\Omega {\phi _i{{{\mathrm{d}}}}{{{\boldsymbol{r}}}}} }}{{V_i\left( t \right)}} - 1} \right){{{\hat{\boldsymbol n}}}}.$$

where *M*′ is the volume constriction strength. By limiting the relative error, the new formulation can achieve a more accurate simulated volume, consistent with the designated one measured experimentally or set arbitrarily. Apart, the stronger volume constriction for the cells with small size ($\sim \!10^2$ ${{{\mathrm{\mu m}}}}^3$) lowers the cell size threshold of cell disappearance. The new formation also maintains numerical stability when handling the relatively larger cells ($10^3\sim 10^4$ ${{{\mathrm{\mu m}}}}^3$), while strengthening volume constriction in the previous formation (i.e., by amplifying *M*) would cause numerical instability easily. The second way to resist cell disappearance is to amplify the value of parameter *c*, the positive coefficient of double-well potential $W(\phi )$, so that the ability of a phase field to separate into two phases can be enhanced (Eq. (1)) (Fig. 3c). Here, we select $c = 2$ and $M\prime = 8$ (with a similar error level to the previous formulation at 4-cell stage) considering both numerical stability and accuracy for simulations of later stages, where the derived binarized relatively strong and weak cell–cell attraction is $\sigma _{{{\mathrm{S}}}} = 0.5$ and $\sigma _{{{\mathrm{W}}}} = 0$ respectively. The final iterative process with the stabilization term added and the volume constriction modified is detailed in Supplementary Note 2.

### A Matlab-based GUI for automatic computation and structural illustration

**Fig. 4:**
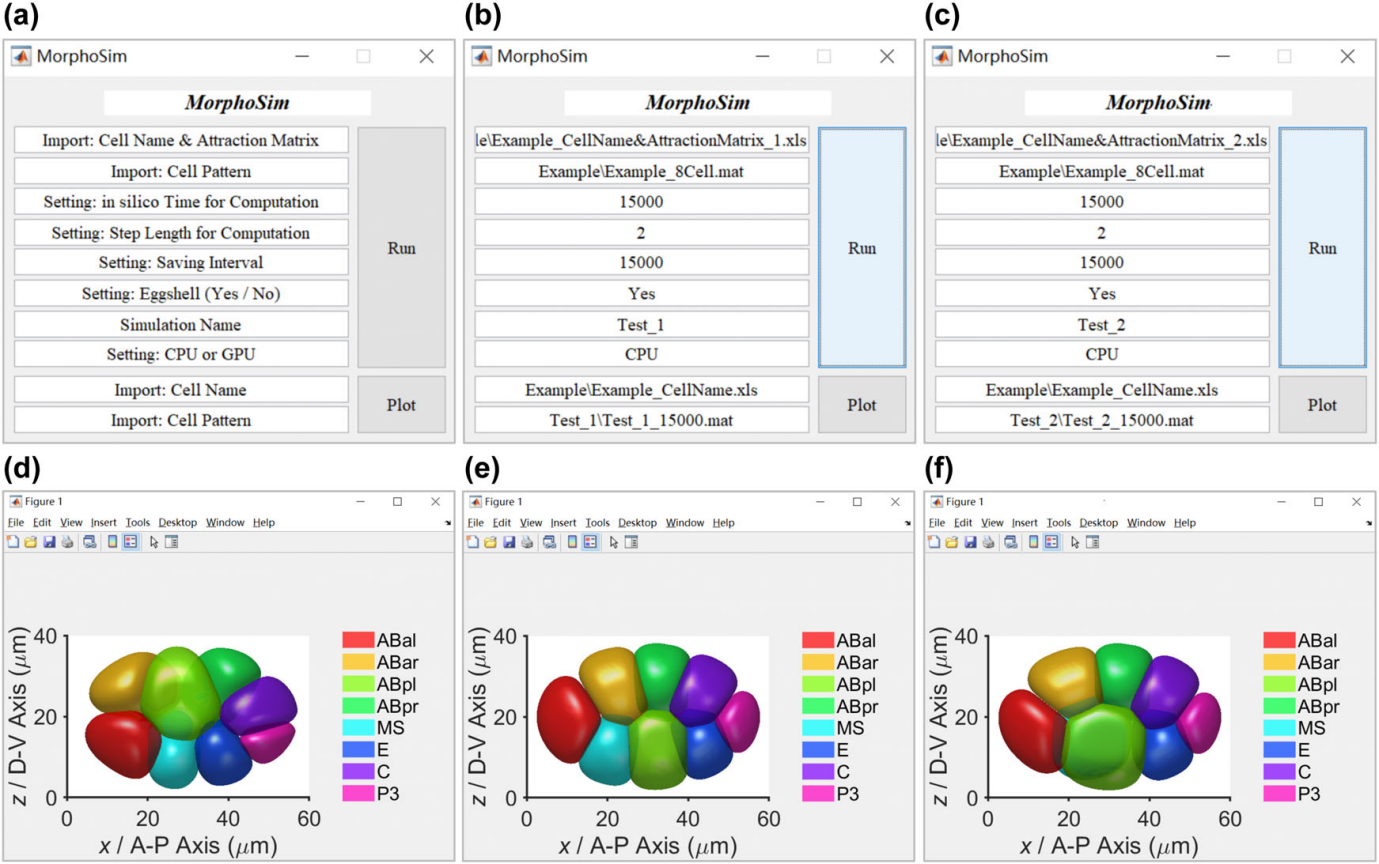
The graphical user interface of *MorphoSim*. **a** The interface and instruction of inputs required. **b**, **c** The simulation inputs for 8-cell *C. elegans* embryogenesis with relatively strong (i.e., $\sigma _{{{{\mathrm{ABpl}}}},{{{\mathrm{E}}}}} = \sigma _{{{\mathrm{S}}}} = 0.5$) and weak (i.e., $\sigma _{{{{\mathrm{ABpl}}}},{{{\mathrm{E}}}}} = \sigma _{{{\mathrm{W}}}} = 0.5$) adhesion in ABpl-E contact respectively. (**d**) The initial state (in silico time = 0) of the 8-cell embryo. **e**, **f** The final state (in silico time = 15,000) of the 8-cell embryo with relatively strong (i.e., $\sigma _{{{{\mathrm{ABpl}}}},{{{\mathrm{E}}}}} = \sigma _{{{\mathrm{S}}}} = 0.5$) and weak (i.e., $\sigma _{{{{\mathrm{ABpl}}}},{{{\mathrm{E}}}}} = \sigma _{{{\mathrm{W}}}} = 0.5$) adhesion in ABpl-E contact respectively.

Given the phase field model and numerical methods, we achieve the final framework named *MorphoSim* (**Morpho**logy **Sim**ulator). We further pack it into an open-source graphical user interface (GUI) using the software Matlab (Fig. 4a)^64^. One can input the binary distribution of cells, assign a cell–cell attraction matrix arbitrarily, and set up the in silico time, step length (i.e., time step size *δt*), and saving interval for simulation. It should be pointed out that the eggshell boundary can be removed as an open-boundary condition and the computation can be performed on a CPU or GPU according to user requirements. When the simulation is over, the user can import the output file of a specific time point and plot the 3D structure automatically. The GUI and a detailed user guidebook are accessible at https://github.com/XiangyuKuang/MorphoSim.

During the 8-cell *C. elegans* embryogenesis, the relatively weak adhesion in ABpl-E contact was reported to be critical for the robust formation of 3D embryonic structure, which serves as a criterion to check if the framework preserves its precision (Fig. 2c)^43^. The inputs for 8-cell simulations with relatively strong (i.e., $\sigma _{{{{\mathrm{ABpl}}}},{{{\mathrm{E}}}}} = \sigma _{{{\mathrm{S}}}} = 0.5$) and weak (i.e., $\sigma _{{{{\mathrm{ABpl}}}},{{{\mathrm{E}}}}} = \sigma _{{{\mathrm{W}}}} = 0$) adhesion in ABpl-E contact are shown in Fig. 4b, c, and the initial state of phase fields is shown in Fig. 4d. The simulation results are in line with previous findings (Fig. 4e, f)^43,56^. Using a personal computer, this simulation lasts for less than 1.5 h on CPU (Intel(R) Core(TM) i5-10210U CPU @ 1.60 GHz 2.11 GHz) and less than 12.5 min on GPU (NVIDIA GeForce GTX 1060) for an in silico time = 15,000.

### Simulation of the *C. elegans* embryogenesis from 1- to 102-cell stages

**Fig. 5:**
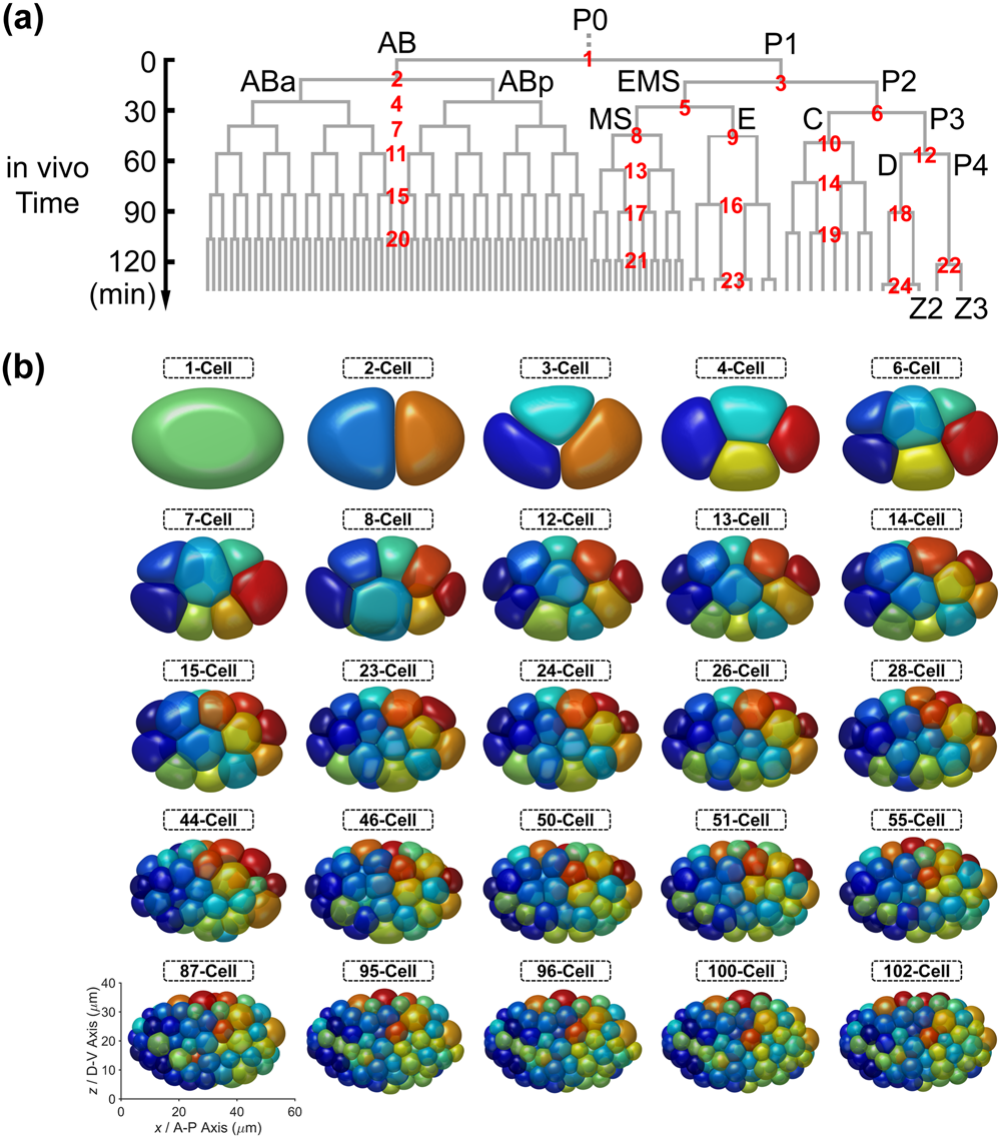
The 1- to 102-cell *C. elegans* embryogenesis. **a** The cell lineage tree reconstituted from in vivo experiment. The cell divisions from the same founder cell (i.e., AB, MS, E, C, D, or P4) and in the same generation are regarded as synchronous; the cell cycle length of each division group is approximated with the shortest one among the cells obtained from experimental measurement^65^. The in vivo time axis is placed on the left and the cell division order is labeled near each cell division group. **b** The embryonic morphologies generated by phase-field simulation, using the cell division order and axis and volume segregation ratio measured experimentally as input. At each stage defined by the cell division order in (**a**), the in silico time (i.e., simulation time) from 1- to 8-cell stages is introduced in Supplementary Note 1, while the one after the 8-cell stage is set to reach the equilibrium ($\bar v \,<\, 1 \times 10^{ - 4}$).

To test the availability of *MorphoSim*, we first perform the simulations of *C. elegans* embryo from 1- to over 100-cell stages according to the cell division order observed in vivo (Supplementary Table 1). For simplicity, here we consider the 6 founder cells (i.e., AB, MS, E, C, D, and P4) as well as their ancestors (i.e., P0, P1, P2, P3, and EMS) and progenies (Fig. 5a). The cell divisions in the same generation of a founder cell are pseudo-synchronous with a slight variation in reality and are idealized as a whole division group with the same cell cycle length in simulation. We use the experimentally-measured shortest cell cycle length within the group as the common value^65^; finally, we obtain the cell division order formed by 24 independent division groups as shown in Fig. 5a. Besides, the inputted cell division axis and volume segregation ratio are acquired from^48^ (Supplementary Table 1). The simulation from 1- to 8-cell stages is performed following the pipeline in Supplementary Note 1, while the one after the 8-cell stage is set to reach mechanical equilibrium (defined by $\bar v \,<\, 1 \times 10^{ - 4}$) or last for a long enough duration (set as in silico time = 10,000) at each stage and then the next cell division(s) would be activated; after 8-cell stage, the cell–cell attraction matrix is still simplified as binary and follows the rule that the attraction between non-sister cells and between sister cells is relatively strong ($\sigma _{{{\mathrm{S}}}} = 0.5$) and weak ($\sigma _{{{\mathrm{W}}}} = 0$) respectively^43^. With the inputs above and the time step size $\delta t = 1.5$, the phase-field framework successfully simulates a multicellular system with up to 102 cells, without any cell disappearance (Fig. 5b and Supplementary Movie 2).

Next, we test its computational efficiency in GPU and CPU compared to the previous phase-field framework, in which the in silico time scale has been well fitted with the in vivo one^43^. Here, we perform the simulation from 6- to 8-cell stages with the previous and current frameworks, whose time step size *δt* is 0.1 and 2 respectively (Fig. 2b, c)^43^. The time scales between the two frameworks are proportionally fitted using the duration of 6- and 7-cell stages determined by their first quasi-steady states so that the simulation process of each stage is equivalent in both frameworks and is comparable with the in vivo time scale. It shows that *MorphoSim* reaches a computational efficiency of more than 10^2^ times the previous one averagely, where the computing time in GPU is around the 0.1- to 1-min levels (Table 1). Besides, for all the 6-, 7-, and 8-cell stages, the mean positional shift of the cells at each time step *δt* in *MorphoSim* simulation is at least one magnitude larger than the one in the previous framework (Supplementary Fig. 6). Further, we increase the cell number to 25, 50, and 100 and estimate the time cost for the system to evolve for an in silico time corresponding to 5 min in reality. For the original framework, the computation is out of memory in both GPU and CPU when the cell number reaches 100. The problem is well solved in *MorphoSim*, allowing full utilization of computational resources and numerical experiments coupled with parameter scanning (Table 1).

### Reconstruction of the assembly, self-repairing, and dissociation of the *synNotch* systems

**Fig. 6:**
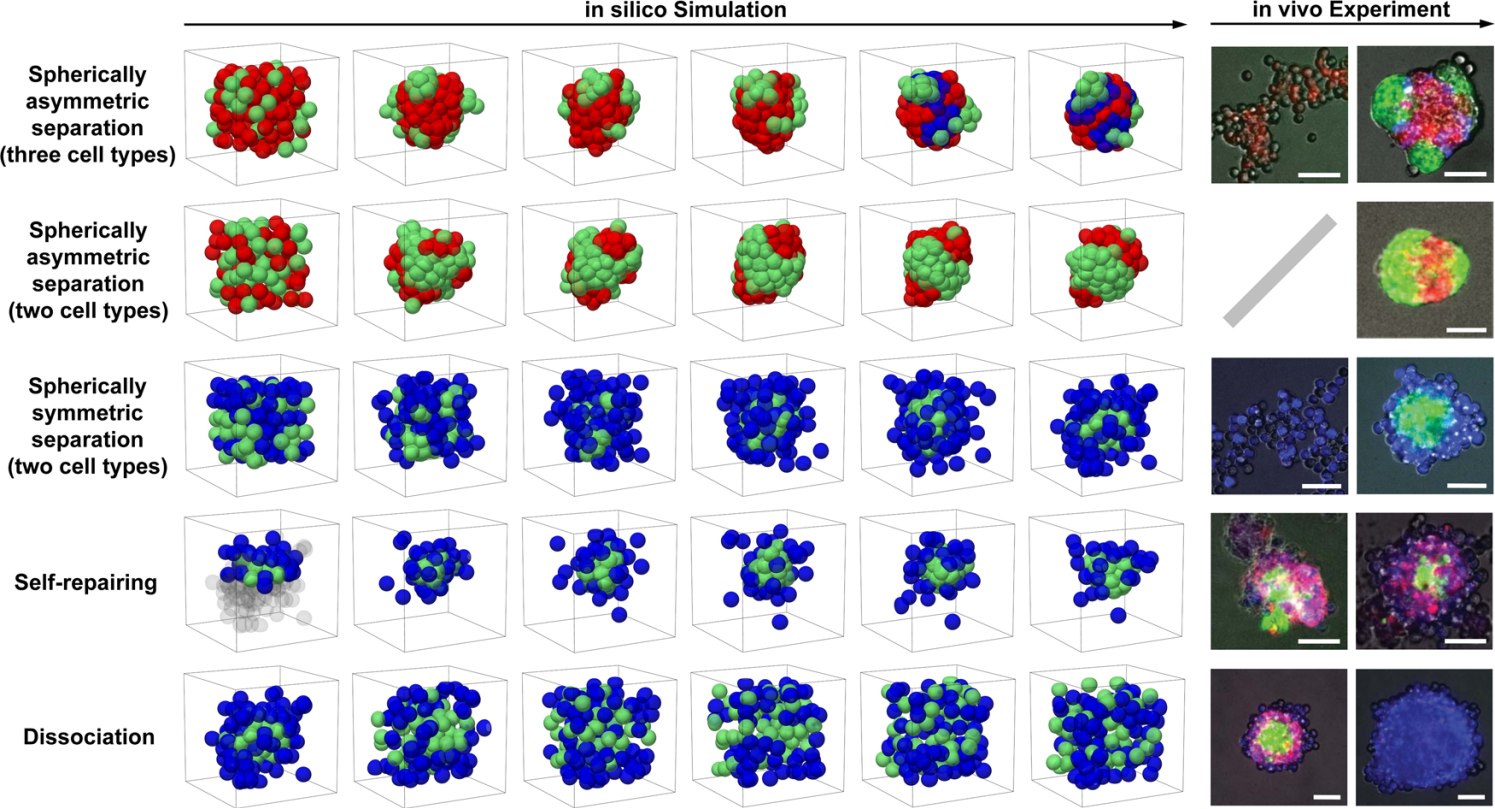
In silico simulation (left panel) and in vivo experiment (right panel) of the *synNotch* systems. In each row, the in silico structures are shown from time points 0 to 50,000 in a step of 10,000; the computational domain is outlined with a black cube. In the first row, since in silico time = 40,000 (the fifth column), a part of red cells, which contact at least one green cell, are painted blue considering that the red cells (Type 1) receive contact-dependent signaling from the green cells (Type 2) and undergo subsequent differentiation into the blue cells (Type 3), in accordance to the experimental description^16^. In both the fourth and fifth rows, the first in silico structure is adopted from the last one in the third row; for the former one, the cells with *z* < 0 or without contact with the cell aggregate are removed and shown by gray shadow. For the in silico structures, the cell types and corresponding colors and value assignments on cell–cell adhesion are listed in Table 2. For the in vivo structures, the scale bars represent 100 μm in reality; the fluorescence colors are in line with the ones used for the in silico structures, except that the red cells in the fourth and fifth rows also correspond to the blue ones in silico as they are the same in the cell–cell adhesion program. The images of in vivo experiment are from ref. ^16^, reprinted with permission from AAAS.

The *synNotch* system is the state-of-the-art methodology to generate self-organizing multicellular living machines, which consist of multiple cell types (illustrated by different colors in Fig. 6) with genetically programmed differential adhesion^16^. The topology of cell aggregate is dependent on the combinatorial cell–cell adhesion strengths and can self-repair after cleavage and dissociate after eliminating the adhesive protein. Here, we employ *MorphoSim* to reproduce the stereotypic tricomponent and biocomponent topologies reported before, which can form spherically asymmetric and symmetric patterns corresponding to two different sets of cell–cell adhesion programs.

In the simulation of the *synNotch* systems, we add the Gaussian white noise ${{{\boldsymbol{\xi }}}}_i\left( t \right)$ onto each cell’s motion to model the stochasticity in reality. Hence, the evolution equation turns into:

13 $$\frac{{\partial \phi _i}}{{\partial t}} = - \frac{1}{\tau }\left( {{{{\boldsymbol{F}}}}_{{{{\mathrm{ten}}}}} + {{{\boldsymbol{F}}}}_{{{{\mathrm{vol}}}}} + {{{\boldsymbol{F}}}}_{{{{\mathrm{rep}}}}} + {{{\boldsymbol{F}}}}_{{{{\mathrm{atr}}}}} + \kappa {{{\boldsymbol{\xi }}}}_i\left( t \right)} \right) \cdot \nabla \phi _i,$$

where *κ* is the noise strength. For all the simulations, the computational domain is set as a 128 × 128 × 128 cubic grid with grid size $\delta l = 0.5$ ${{{\mathrm{\mu m}}}}$ and time step size $\delta t = 1.25$, and *κ* is assigned $\frac{{\sqrt 5 }}{{10}}$. It’s worth noting that the spatial scale of the simulation can be adjusted to the experimental one by rescaling parameters. Here we provide a rescaled parameter setting with the spatial scale of simulations approximating the experimental scales of the *synNotch* system (Supplementary Table 5)^16^. To set up the initial state, we uniformly randomize the cell positions inside the cubic domain, while each cell is assigned a sphere with a radius $R = 5$ ${{{\mathrm{\mu m}}}}$; then we impose an intercellular repulsion ${{{\boldsymbol{F}}}}_{i,j}$ and a repulsion ${{{\boldsymbol{F}}}}_{{{{\mathrm{boundary}}}},i}$ between cell and boundary to eliminate the possible overlap between cellular regions:

14 $$\boldsymbol{F}_{i,j} = \left\{\begin{array}{cc} k_1\left(\bar{\boldsymbol r}_i-\bar{\boldsymbol r}_j\right),&\left|\bar{\boldsymbol r}_i-\bar{\boldsymbol r}_j\right|\,<\,2R\\ \mathbf {0},&\left|\bar{\boldsymbol r}_i-\bar{\boldsymbol r}_j\right|\,\ge\,2R\end{array}\right.,$$

15 $${{\boldsymbol{F}}_{{\rm{boundary}},i}} = \left\{ {\begin{array}{*{20}{c}}{{k_2}({{\boldsymbol{d}}_{{\rm{boundary}},i}}-R),}&{|{{\boldsymbol{d}}_{{\rm{boundary}},i}}|\, < \,R}\\{{\mathbf{0}},}&{|{{\boldsymbol{d}}_{{\rm{boundary}},i}}| \ge R}\end{array}.} \right.$$

Here, ${{{\bar{\boldsymbol r}}}}_i$ and ${{{\bar{\boldsymbol r}}}}_j$ are the centroids of the *i*-th and *j*-th cells; ${{{\boldsymbol{d}}}}_{{{{\mathrm{boundary}}}},i} = \left( {d_{i,x},d_{i,y},d_{i,z}} \right) = {{{\mathrm{min}}}}\left( {L - {{{\bar{\boldsymbol r}}}}_i,{{{\bar{\boldsymbol r}}}}_i} \right)$ is the nearest distance between the centroid of the *i*-th cell and the cubic boundary in three orthogonal directions; *L* is the side length of the cubic domain; $k_1 = 0.05$; $k_2 = 0.1$. The iteration ${{{\bar{\boldsymbol r}}}}_i^{n + 1} = {{{\bar{\boldsymbol r}}}}_i^n + {{{\boldsymbol{F}}}}_{{{{\mathrm{boundary}}}},i} + \mathop {\sum }\nolimits_{i \ne j} {{{\boldsymbol{F}}}}_{i,j}$ continues until all the cells have a distance no less than 2*R* from the others (roughly 200–300 time steps), then their distribution will be used as the initial condition for the *synNotch* simulation. The simulation scenarios along with their parameter settings and time scales are described in Table 2, where all the simulations last for an in silico time = 50,000 and no cell division is executed. It is noteworthy that the adhesion within and between cell types is set once for all and only the simulation for the tricomponent spherically asymmetric separation incorporates cell differentiation, described by a cell type transition since in silico time = 40,000 based on contact-dependent cell–cell signaling.

**Table 2:** Parameter settings and time scales of the *synNotch* simulations.

Topological dynamic	Stage	Cell type 1^a^	Cell type 2^a^	Cell type 3^a^	In silico time	Computing time (h)	Value assignment on cell–cell adhesion
Spherically asymmetric separation (three cell types)^b^	Start	80 (R)	40 (G)	0 (B)	50,000	12.2	*σ*_1,1_ = 0.9
	End	40 (R)	40 (G)	40 (B)			*σ*_1,2_ = 0.5
Spherically asymmetric separation (two cell types)	From start to end	62 (R)	62 (G)	/	50,000	11.9	*σ*_2,2_ = 0.9
Spherically symmetric separation		50 (G)	75 (B)	/	50,000	13.3	*σ*_1,1_ = 0.9
Self-repairing		24 (G)	32 (B)	/	50,000	4.4	*σ*_1,2_ = 0.5
Dissociation		50 (G)	75 (B)	/	50,000	12.1	*σ*_2,2_ = 0.3

^a^The painting color of each cell type in Fig. 6 is labeled in the columns “Cell type 1”, “Cell type 2”, and “Cell type 3”, where “R”, “G”, and “B” represent red, green, and blue, respectively.

^b^For the “Spherically asymmetric separation (three cell types)”, “Cell type 3” is defined as the cell that originally belongs to “Cell type 1” but contacts at least one cell in “Cell type 2” since in silico time = 40,000, so the mechanical properties of “Cell type 3” is same to the ones of “Cell type 1”.

The first condition is to program two types of cells, which have strong adhesion within either cell type and weak adhesion between the two cell types (Table 2). Since the cells of either cell type tend to be near each other, the system would separate into two major regions corresponding to the two cell types (second row in Fig. 6 and Supplementary Movie 3). If a cell in Type 1 is additionally programmed to differentiate into another fate (Type 3) after a specific time point (i.e., in silico time = 40,000) but maintains its original adhesion properties when contacting a cell in Type 2, a nested structure formed by three cell types appears as seen in vivo (first row in Fig. 6 and Supplementary Movie 4). The second condition is to program two types of cells as well, but the cells in Type 1 and Type 2 have strong and weak adhesion respectively (Table 2), which generates a layered structure in line with the experimental one (third row in Fig. 6 and Supplementary Movie 5). Furthermore, we adopt the final state of the layered structure and test if the self-repairing (by removal of half of the cells) and dissociation (by eliminating cell–cell adhesion) take place when the experimental conditions are mimicked in silico. As expected, both morphogenetic phenomena are successfully reproduced in simulation (fourth and fifth rows in Fig. 6 and Supplementary Movies 6, 7).

### Simulating and exploring more biological processes extensively with *MorphoSim*

As exemplified by simulations on the nematode embryogenesis and *synNotch* systems, *MorphoSim* is capable of modeling organic and embryonic morphologies precisely. Despite that only the cell–cell attraction is studied as a variable, all physical parameters can be changed and adapted to specific biological processes. The key physical parameters and their biological significance are listed in Table 3, in which we explain how they should be customized for different biological scenes.

**Table 3:** The key physical parameters and their biological significance.

Physical parameter	Biological significance	Remark
*γ*	Cell surface tension	The cell surface tension is mainly attributed to cortical contractility^72^. As cell surface tension minimizes the cell surface area and pushes a cell to be spherical instead of being deformed, it also characterizes the effect of cell stiffness (hard or soft) if the cell’s natural shape is spherical (Supplementary Fig. 7a–c and Supplementary Table S6). In reality, the cell stiffness is contributed by the cytoskeleton and nucleus and varies from cell type to cell type. For instance, the tumor cells are usually softer than their coexisting benign counterparts^42,73,74^.
$\phi _{{{\mathrm{e}}}}$	Eggshell/boundary	The size and shape of the eggshell or boundary can be customized according to the system of interest (e.g., ellipsoidal or cylindroid for *C. elegans* embryo and boundless for *synNotch* system)^16,52,53^.
*σ*	Cell–cell attraction	The attraction between cells often comes from cell–cell adhesion (e.g., cadherin family), gap junction (e.g., connexin family), and ligand-receptor binding^75–77^. Asymmetric intercellular attraction ($\sigma _{i,j} \ne \sigma _{j,i}$) could be employed to model the biological phenomena in which one cell is unidirectionally attracted by another, such as the chemotactic cell movement induced by cell–cell signaling and engulfment during apoptosis^78–80^.
$V_i\left( t \right)$	Cell volume	The cell volume can be time-dependent according to specific situations. For example, it decreases during cell apoptosis and increases during cell growth^63,81^. The cell volume can also be affected by extrinsic factors like osmotic pressure and substrate stiffness^82,83^.
*τ*	Ambient viscosity	The viscosity is dependent on the real scenario^84^. When the environment disobeys the overdamping condition, one can rewrite Eq. (5) accordingly^85,86^.
*κ*	Stochastic noise	The stochasticity in cell movement can be raised by intrinsic (e.g., thermal effect and fluctuation) and extrinsic (e.g., stirring during incubation of the *synNotch* spheroid) reasons^16,87,88^.

## Discussion

Multicellular morphology is a fascinating topic and a long-term focus of biological research. Extending on our previous phase field model established with high-quality in vivo data^43,48^, in this work we developed an efficient and scalable framework, *MorphoSim*, for multicellular systems. We proposed a stabilized numerical scheme and new volume constriction to lower the model’s computational cost for simulating a large number of cells simultaneously with considerable accuracy. *MorphoSim* provides an efficient and powerful tool that not only affords large-scale simulations, but also allows high-dimensional parameter scanning on GPU/CPU clusters. Moreover, the morphodynamics of the tricomponent and biocomponent *synNotch* systems can be reproduced by *MorphoSim* in approximately half a day, demonstrating the framework’s validity, efficiency, and applicability.

In synthetic biology, more and more attentions are being paid to cell-based morphology synthesis including organoid, embryoid, bio-robot, etc. Taking the *synNotch* system as an example, they are genetically programmed with differential cell–cell adhesion to achieve different modes of structure and function. However, it still faces some challenges: e.g., (1) How do biological parameters like cell stiffness and surface tension affect the dynamics? (Supplementary Fig. 7a–c and Supplementary Table 6); and (2) How to choose the parameter combination to optimize a specific function? It would be very helpful if the parameter space can be efficiently explored^66^. *MorphoSim* can regenerate the *synNotch* dynamics quickly and large-scale simulations can also be carried out on GPU and CPU clusters for both mechanistic studies and parameter optimization. Note that the *MorphoSim* parameters (e.g., adhesion strength, motional noise, cell number, spatial resolution, and temporal resolution) should be readjusted to the real experimental condition for better simulation performance.

The proposed phase-field framework is also practical to model natural systems such as a developing embryo with large cell numbers. By comparing the in silico and in vivo morphologies, the low-cost parameter scanning permits the inference of a multicellular system’s mechanical state, which is hard to measure directly or infer by the previous phase field model^43^. As the phase field model describes a cell on a dense mesh, more space-related biochemical and biophysical details (e.g., polarity and cytoskeleton) can be added to reconstruct the real system comprehensively.

Despite that *MorphoSim* can simulate over one hundred interacting cells, there is still a great need from the field to keep increasing the cell number and decreasing the computational cost. For example, the early *Drosophila* embryogenesis involves thousands of cells and was usually simulated using the coarse-grained model or vertex model^67,68^. On the one hand, the limited GPU memory hampers mesh enlargement/densification when considering a large number of cells. Algorithms like parallel computation^69^, adaptive mesh refinement^70^, and moving mesh method^71^, can be employed to reduce the computational cost and improve efficiency.

In addition to the algorithmic improvements above, the *MorphoSim* framework can be further developed based on the multiscale method. Instead of using a phase field variable to represent a single cell, we may use a macroscopic phase field variable to represent a group of cells that have the same biophysical properties and microscopic phase field variables to mimic the cells with distinct properties, which could greatly reduce the computational cost and allow for simulations with a much larger number of cells. Besides, for an in vivo system with known morphological information (e.g., *C. elegans* embryo)^48^, it is feasible to only simulate a special group of cells or the regions of interest instead of the entire system while assigning the cell morphology measured experimentally onto the other cells as a boundary constraint.

## Supplementary information

Supplementary Information

Supplementary Movie 1

Supplementary Movie 2

Supplementary Movie 3

Supplementary Movie 4

Supplementary Movie 5

Supplementary Movie 6

Supplementary Movie 7

## Data Availability

All relevant data are within the paper and its Supplementary Information files.

## Code availability

The scripts of *MorphoSim* and its Matlab-based GUI are deposited at https://github.com/XiangyuKuang/MorphoSim. In addition to *MorphoSim*, all other source codes used in this paper are available on reasonable request.
