# Supplementary material for: MorphoSim: an efficient and scalable phase-field framework for accurately simulating multicellular morphologies
Source: NPJ Syst Biol Appl. 2023 Feb 17;9:6. doi: 10.1038/s41540-023-00265-w (PMC9938209; doi:10.1038/s41540-023-00265-w)
Supplement: Supplementary file 1 — Supplementary Information [file 41540_2023_265_MOESM1_ESM.pdf]

Supplementary Information

MorphoSim: an efficient and scalable phase-field framework for accurately simulating multicellular morphologies

Xiangyu Kuang[1†], Guoye Guan[1†], Chao Tang[1,2,3], Lei Zhang[1,4,5*]*

[1] *Center for Quantitative Biology, Peking University, Beijing 100871, China*

[2] *Peking-Tsinghua Center for Life Sciences, Peking University, Beijing 100871, China*

[3] *School of Physics, Peking University, Beijing 100871, China*

[4] *Beijing International Center for Mathematical Research, Peking University, Beijing 100871, China*

[5] *Center for Machine Learning Research, Peking University, Beijing 100871, China*

[†] *These authors contributed equally to this work.*

[*] *For correspondence: tangc@pku.edu.cn (CT), zhangl@math.pku.edu.cn (LZ)*

Supplementary Figures

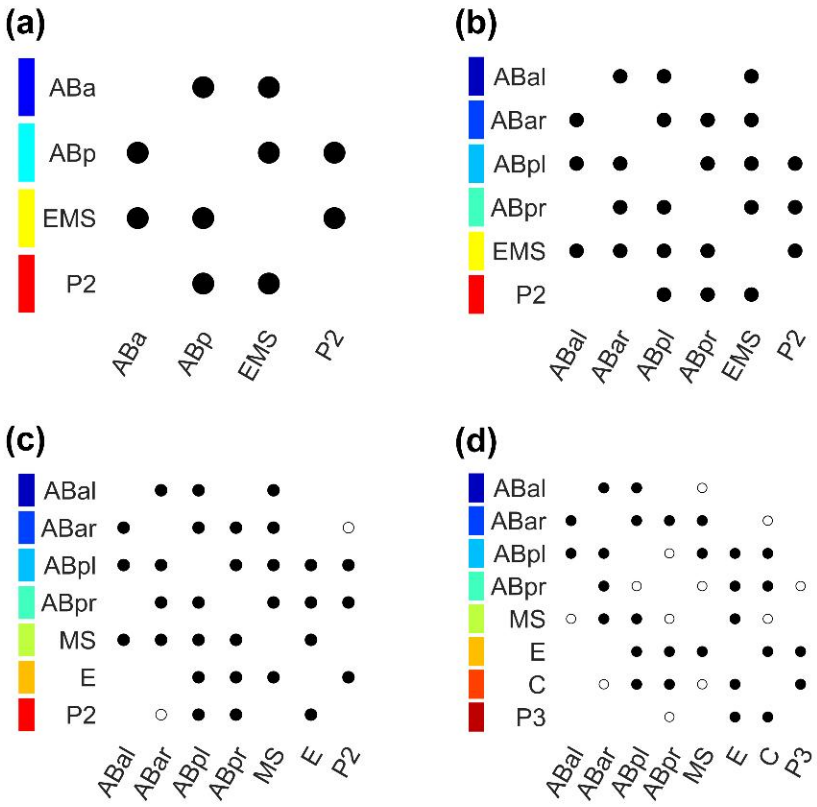

Supplementary Figure 1. Cell-cell contact map of *in vivo C. elegans* embryos at (a) 4-cell, (b) 6-cell, (c) 7-cell, and (d) 8-cell stages. Solid and empty circles denote the conserved and unconserved cell-cell contact pairs, while the remaining blank regions mean that no contact is found between the corresponding cell pair. The contact information is summarized using 17 embryo samples at their last imaging time points of each stage, where the cells undergoing cytokinesis (i.e., with two nuclei embedded by one membrane) are all excluded [1].

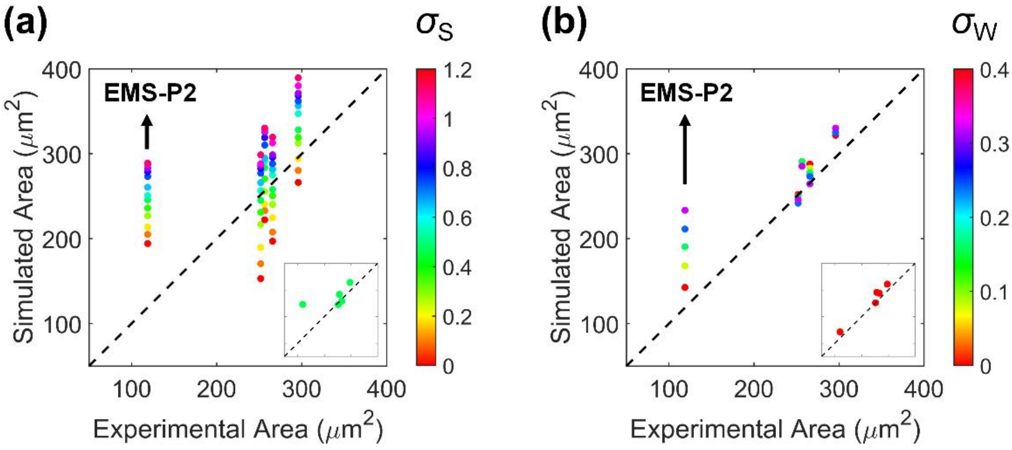

Supplementary Figure 2. Fitting between simulated and experimental cell-cell contact areas at 4-cell stage by tuning the relatively strong (σ_S) and weak (σ_W) cell-cell attraction strength. (a) Simulation with relatively strong attraction σ_S assigned on all the 5 cell-cell contacts and varied from 0 to 1.2 in a step of 0.1, showing the optimal value assignment as $\sigma_S = 0.5$ (highlighted in the inset). (b) Simulation with relatively weak attraction σ_W assigned on the EMS-P2 contact and varied from 0 to 0.4 in a step of 0.1 and relatively strong attraction $\sigma_S = 0.5$ assigned on the other 4 cell-cell contacts, showing the optimal value assignment as $\sigma_W = 0$ (highlighted in the inset). The details for cell-cell contact area fitting are introduced in Supplementary Note 1.

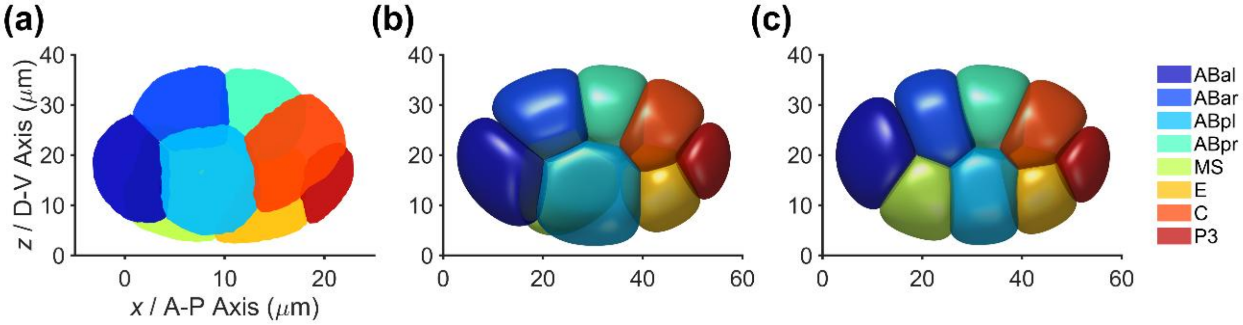

Supplementary Figure 3. *C. elegans* embryonic morphology at 8-cell stage. (a) The typical 8-cell *C. elegans* morphology *in vivo*, with its cell-cell contact map shown in Supplementary Fig. 1d. (b) The simulated 8-cell *C. elegans* morphology with relatively weak attraction in ABpl-E contact (i.e., $\sigma_{\mathrm{ABpl,E}} = \sigma_W$) when the 8 cells have moved for an *in silico* time = 15000. The multicellular structure remains three-dimensional and there's no change in its cell-cell contact map. (c) The simulated 8-cell *C. elegans* morphology with relatively strong attraction in ABpl-E contact (i.e., $\sigma_{\mathrm{ABpl,E}} = \sigma_S$) when the 8 cells have moved for an *in silico* time = 15000. The multicellular structure collapses into an incorrect structure much earlier than the one with relatively weak attraction. The value assignments on relatively strong and weak attraction (i.e., σ_S and σ_W) are fitted with the cell-cell contact areas at 4-cell stage, as introduced in Supplementary Note 1 and Supplementary Fig. 2a, b. The simulation in (b)(c) is performed using the original phase-field framework ($\delta l = 0.25$ μm, $\delta t = 0.1$) [2], where the fitted cell-cell attraction is $\sigma_S = 0.9$ and $\sigma_W = 0.2$.

(a)

Simulation Procedure	Requirement	Explanatory Figure
1- to 3-Cell Stages (without attraction)	● The computation is stable.<br>● ABa contacts with P1.	Fig. 1b
4-Cell Stage (without attraction)	● All the 5 cell-cell contacts are reproduced.<br>● The anterior-posterior and dorsal-ventral axes are established.<br>● All contact areas are smaller than the experimental values.<br>***Significance: Indicating the existence of cell adhesion***	Fig. 1b<br>Supplementary Fig. 1a
4-Cell Stage (fitting of global attraction)	● The contact areas are larger than the ones simulated without attraction.<br>● The contact areas except EMS-P2 are near the experimental values.<br>● The contact area of EMS-P2 is larger than the experimental value.<br>***Significance: Indicating the relatively weak adhesion in EMS-P2 contact***	Supplementary Fig. 2a
4-Cell Stage (fitting of local attraction)	● The attraction of EMS-P2 contact is weaker than the global one.<br>● All contact areas are near the experimental values.	Supplementary Fig. 2b
6- to 8-Cell Stages	● The conserved cell-cell contact maps at 6- and 7-cell stages are reproduced.<br>● The conserved cell-cell contact map at 8-cell stage are reproduced stably in the simulation with relatively weak attraction in ABpl-E contact, but not in the one with relatively strong attraction.<br>***Significance: Indicating the relatively weak adhesion in ABpl-E contact***	Supplementary Fig. 1b-d<br>Supplementary Fig. 3a-c

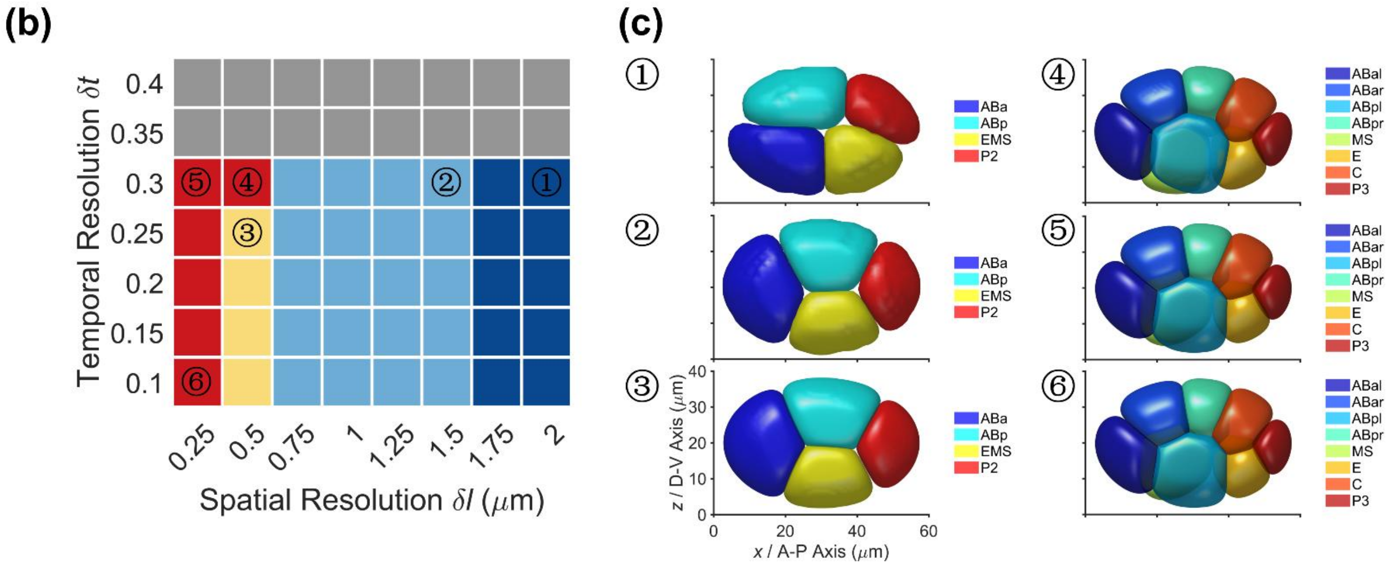

Supplementary Figure 4. Framework improvement by refinement of spatial and temporal resolutions. (a) Quality-control pipeline to verify if a set of simulations capture the *in vivo* system with enough accuracy. The quantitative criteria for each requirement are introduced in Supplementary Note 1. (b) Simulation results under different spatial and temporal resolutions. Each color represents the stage when the simulation dissatisfies the requirements as shown in (a), except that the red means the simulation passes all the requirements. (c) Final embryonic morphologies under the combinations of spatial and temporal resolutions ① ~ ⑥ labeled in (b).

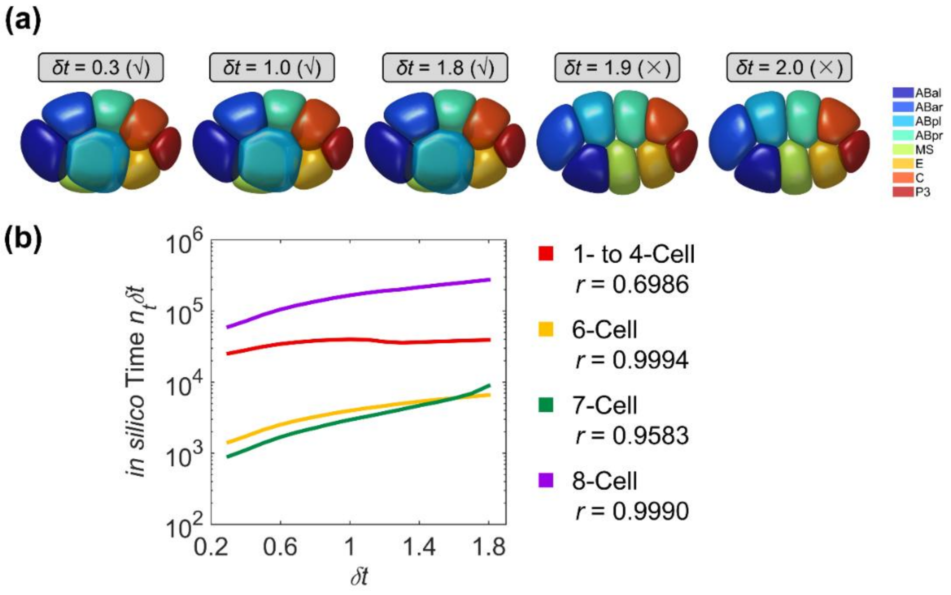

Supplementary Figure 5. Limitation of the framework with first-order stabilization term. (a) Embryonic morphologies at the end of 8-cell stage under δt = 0.3, 1.0, 1.8, 1.9, and 2.0. The ones that successfully achieve the typical structure *in vivo* are labeled with "√" while the others are labeled with "×". (b) *In silico* time $n_t\delta t$ at different stages against time step size δt. The cell stages and corresponding colors and correlation coefficients r ($r = \frac{\sum_{i=1}^{N} X_i Y_i - \frac{1}{N}\sum_{i=1}^{N} X_i \cdot \sum_{i=1}^{N} Y_i}{\sqrt{\left[\sum_{i=1}^{N} X_i^2 - \frac{1}{N}\left(\sum_{i=1}^{N} X_i\right)^2\right]\left[\sum_{i=1}^{N} Y_i^2 - \frac{1}{N}\left(\sum_{i=1}^{N} Y_i\right)^2\right]}}$, where X_i and Y_i are the horizontal and vertical coordinates of the i-th point among N samples) between δt and logarithmic $n_t\delta t$ are listed on right.

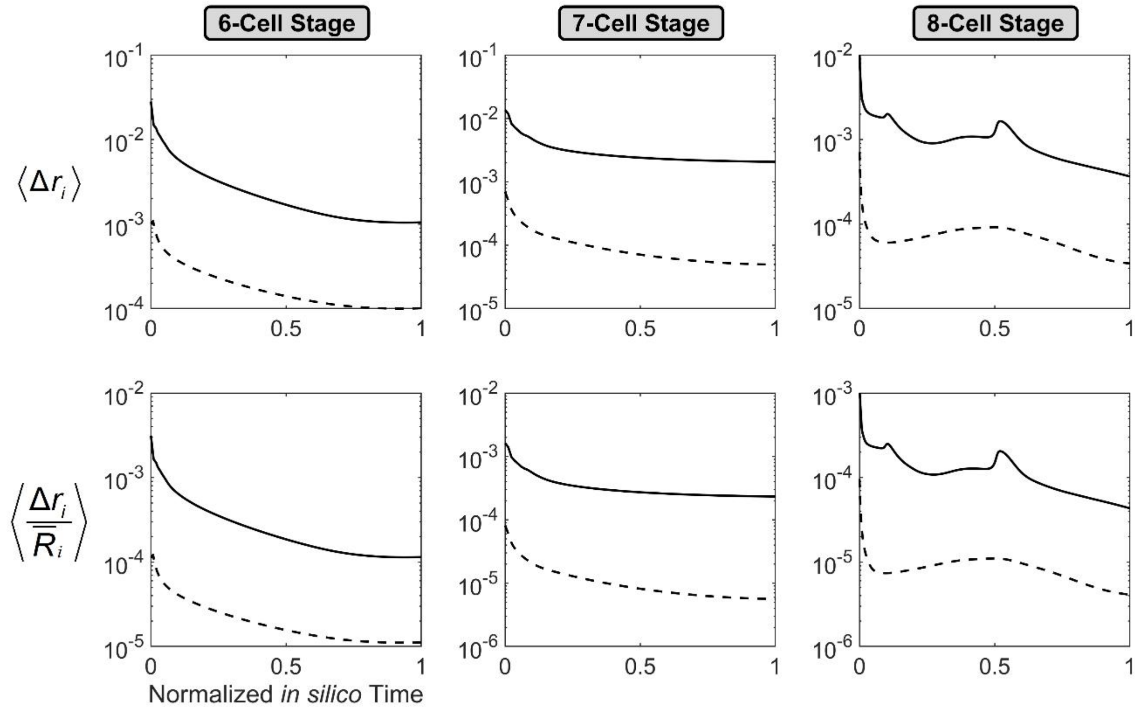

Supplementary Figure 6. The mean positional shift of the cells at each time step in phase-field simulation. The absolute positional shift on average (i.e., $\langle \Delta r_i \rangle$) at 6, 7-, and 8-cell stages (from left to right) are shown in the upper row; the relative positional shift on average (i.e., $\langle \frac{\Delta r_i}{\overline{R}_i} \rangle$) defined by the ratio of the absolute positional shift (Δr_i) to equivalent cell radius ($\overline{R}_i = \sqrt[3]{\frac{3V_i}{4\pi}}$) at 6-, 7-, and 8-cell stages (from left to right) are shown in the lower row; here, Δr_i represents the absolute positional shift of the i-th cell at each time step in phase-field simulation. In all the subfigures, the data in the previous and current frameworks are shown with a dashed line and a solid line respectively; the time step size δt used in the previous and current frameworks is 0.1 and 2 respectively; the *in silico* time (i.e., simulation time) for each stage in both frameworks is introduced in Supplementary Note 1 and is normalized to 0 ~ 1.

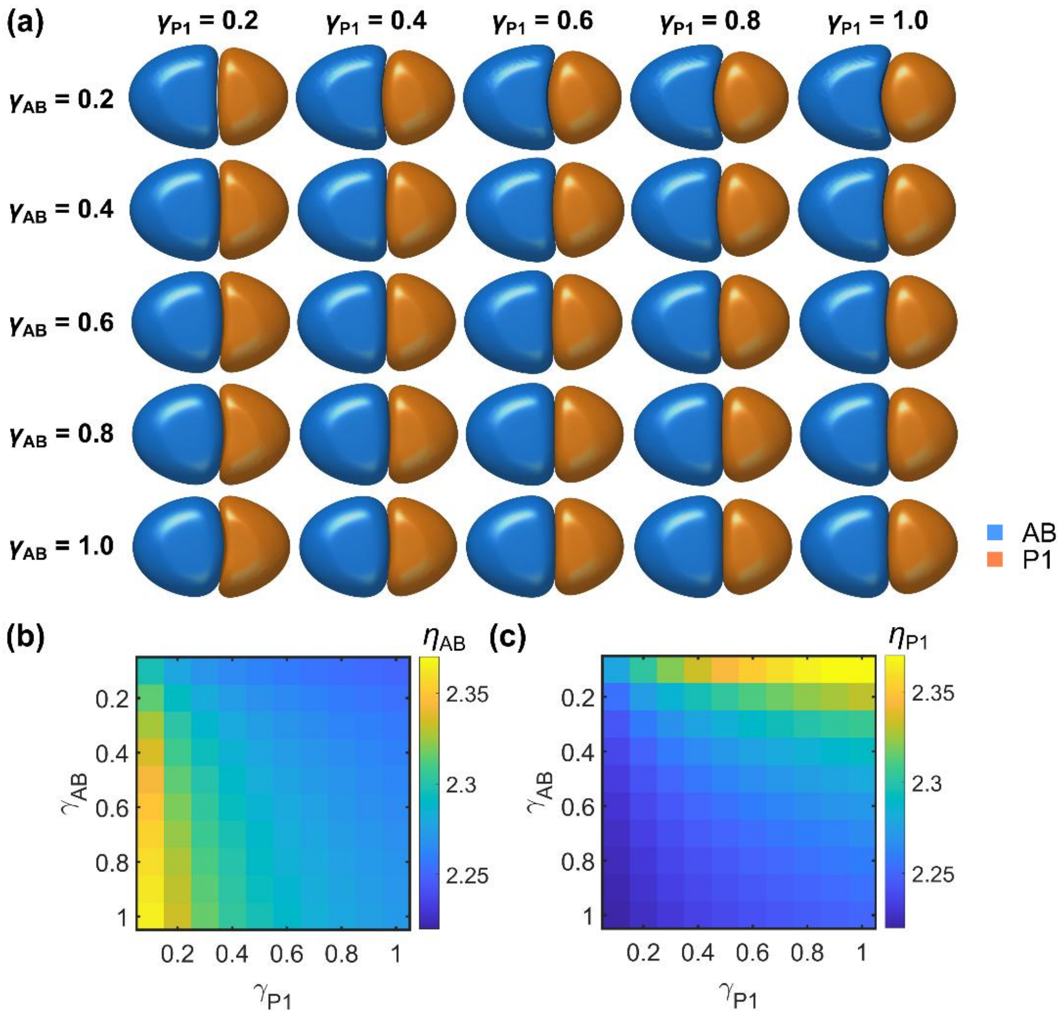

Supplementary Figure 7. The morphological effect of γ that characterizes both cell surface tension and cell stiffness, illustrated by the 2-cell stage with different value assignments. (a) Morphology of AB (blue) and P1 (orange) cells. (b)(c) The irregularity score $\eta = \frac{Cell\,Surface\,Area^{\frac{1}{2}}}{Cell\,Volume^{\frac{1}{3}}}$ of AB and P1 cells respectively [1].

Supplementary Note 1

Search of the minimal spatial and temporal resolutions enough for reproducing the previous findings

Here, we attempt to minimize the spatial and temporal resolutions while guaranteeing the results that are consistent with the ones in [2]. The step-by-step simulation procedure and key biological findings of the original framework are summarized in Supplementary Fig. 4a. With the parameter assignments adopted from [2] and the cell division order and axis and volume segregation ratio acquired from [1] (Supplementary Table 1 and Supplementary Table 2), there are three standards for evaluating if the simulation outputs keep sufficient precision during the 1- to 8-cell *C. elegans* embryogenesis: 1. the cell-cell contact maps include all the contacts and non-contacts that are conserved among individual embryos (Supplementary Fig. 1a-d) [1]; 2. the relatively weak attraction in EMS-P2 and ABpl-E contacts, which has been demonstrated by experimental observation before [3,4], is inferable by morphological comparison between simulation and experiment (Supplementary Fig. 2a, b and Supplementary Fig. 3a-c); 3. the simulated embryonic morphologies match the experimental ones (Fig. 1b) [1]. The quantitative criteria and search pipeline are introduced detailly below.

1. Grid setting

We predetermine the computational domain Ω as a cuboid with a size of $\left(L_x, L_y, L_z\right) = (60\ \mu\text{m}, 30\ \mu\text{m}, 40\ \mu\text{m})$. Then, we consider the scanning range of spatial grid size (spatial resolution) as $\delta l = 0.25\ \mu\text{m}: 0.25\ \mu\text{m}: 2.00\ \mu\text{m}$ and time step size (temporal resolution) as $\delta t = 0.10: 0.05: 0.40$. For each value of δl, the grid nodes in three orthogonal directions are $n_x = \text{CEIL}\left(\frac{L_x}{\delta l}\right)$, $n_y = \text{CEIL}\left(\frac{L_y}{\delta l}\right)$, $n_z = \text{CEIL}\left(\frac{L_z}{\delta l}\right)$, where CEIL is the round-up integral function.

2. Definition of steady state

For the i-th cell's phase field $\phi_i(\boldsymbol{r}, t)$ at specific time point t, its centroid is calculated as $\boldsymbol{r}_i^t = \frac{\int_\Omega \boldsymbol{r}\phi_i^t \mathrm{d}\boldsymbol{r}}{\int_\Omega \phi_i^t \mathrm{d}\boldsymbol{r}}$. We use the consecutive $m = \text{FLOOR}\left(\frac{10}{\delta t}\right)$ time steps to calculate its average velocity during this interval, namely, $\boldsymbol{v}_i^t = \frac{\boldsymbol{r}_i^t - \boldsymbol{r}_i^{t-m\delta t}}{m\delta t}$, where FLOOR is the round-down integral function. When the root-mean-square velocity of all N cells inside the eggshell, $\overline{v^t} = \sqrt{\frac{1}{N}\sum_{i=1}^{N} |\boldsymbol{v}_i^t|^2}$, is smaller than 1×10^{-4}, the whole system is regarded as reaching a steady state or mechanical equilibrium.

3. Regeneration of the key findings in the previous framework

The step-by-step establishment of the original phase-field framework revealed a series of significant biological processes, which are in favorable agreement with *in vivo* observations [2]. The degradation of spatial and temporal resolutions is requested to remain the previously reported key findings unchanged, otherwise, the resolution is considered not precise enough. The simulation procedures (labeled by capital letters) and quantitative criteria (labeled by solid circles) are listed below.

A. Simulating the 1- to 2-cell stages without cell-cell attraction, until reaching the steady state respectively.

- No numeric overflow occurs.

B. Simulating the 3-cell stage without cell-cell attraction, until reaching the steady state.

- The ABa-P1 contact is established.

C. Simulating the 4-cell stage without cell-cell attraction, until reaching the steady state.

- The ABa-ABp, ABa-EMS, ABp-EMS, ABp-P2, EMS-P2 contacts are established (Supplementary Fig. 1a).
- The anterior-posterior and dorsal-ventral axes are established.

$$\boldsymbol{n}_{\mathrm{AP}} = \frac{\boldsymbol{r}_{\mathrm{ABa}} - \boldsymbol{r}_{\mathrm{P2}}}{|\boldsymbol{r}_{\mathrm{ABa}} - \boldsymbol{r}_{\mathrm{P2}}|}, \left|\cos^{-1}\left(\boldsymbol{n}_{\mathrm{AP}} \cdot (1,0,0)\right)\right| < 10°$$

$$\boldsymbol{n}_{\mathrm{DV}} = \frac{\boldsymbol{r}_{\mathrm{ABp}} - \boldsymbol{r}_{\mathrm{EMS}}}{|\boldsymbol{r}_{\mathrm{ABp}} - \boldsymbol{r}_{\mathrm{EMS}}|}, \left|\cos^{-1}\left(\boldsymbol{n}_{\mathrm{DV}} \cdot (0,1,0)\right)\right| < 10°$$

- The areas of 5 contacts are essentially smaller than the real values. Here, we label the simulated and experimental areas of cell-cell contact h with $S_{\mathrm{sim},h}$ and $S_{\mathrm{exp},h}$ respectively.

$$\mathrm{MEAN}\left\{\frac{S_{\mathrm{sim},h} - S_{\mathrm{exp},h}}{S_{\mathrm{exp},h}}\right\} < 0, \text{for all 5 contacts}$$

D. Simulating the 4-cell stage with relatively strong cell-cell attraction $\sigma = 0{:}\,0.1{:}\,1.2$ assigned on all the 5 contacts globally and searching for the optimal σ_{S} value with the smallest $\mathrm{MEAN}\left\{\left|\frac{S_{\mathrm{sim},h} - S_{\mathrm{exp},h}}{S_{\mathrm{exp},h}}\right|\right\}$ for ABa-ABp, ABa-EMS, ABp-EMS, ABp-P2 contacts (Supplementary Fig. 2a).

- The areas of all 5 contacts are enlarged compared to the ones without cell-cell attraction.

$$\mathrm{MEAN}\left\{\left|\frac{S_{\mathrm{sim},h} - S_{\mathrm{exp},h}}{S_{\mathrm{exp},h}}\right|\right\}_{\sigma \neq 0} < \mathrm{MEAN}\left\{\left|\frac{S_{\mathrm{sim},h} - S_{\mathrm{exp},h}}{S_{\mathrm{exp},h}}\right|\right\}_{\sigma = 0}, \text{for all 5 contacts}$$

- The areas of ABa-ABp, ABa-EMS, ABp-EMS, ABp-P2 contacts are close to the real values.

$$\left|\frac{S_{\mathrm{sim},h} - S_{\mathrm{exp},h}}{S_{\mathrm{exp},h}}\right| < 0.20, \text{for ABa} - \text{ABp, ABa} - \text{EMS, ABp} - \text{EMS, ABp} - \text{P2 contacts}$$

- The area of EMS-P2 contact is essentially larger than the real value.

$$\frac{S_{\mathrm{sim},h} - S_{\mathrm{exp},h}}{S_{\mathrm{exp},h}} > 0.40, \text{for EMS} - \text{P2 contact}$$

E. Simulating the 4-cell stage with relatively weak cell-cell attraction $\sigma = 0{:}\,0.1{:}\,(\sigma_{\mathrm{S}} - 0.1)$ specified for EMS-P2 contact locally and σ_{S} assigned on the other 4 contacts, and searching for the optimal σ_{W} value with the smallest $\mathrm{MEAN}\left\{\left|\frac{S_{\mathrm{sim},h} - S_{\mathrm{exp},h}}{S_{\mathrm{exp},h}}\right|\right\}$ for all 5 contacts (Supplementary Fig. 2b).

- The areas of all 5 contacts are close to the real values.

$$\left|\frac{S_{\mathrm{sim},h} - S_{\mathrm{exp},h}}{S_{\mathrm{exp},h}}\right| < 0.20, \text{for all 5 contacts}$$

D. Simulating the 6-cell stage to its quasi-steady state $\left(\left.\frac{\mathrm{d}\bar{v}}{\mathrm{d}t}\right|_{t=t_{\mathrm{q}}} = 0,\ \left.\frac{\mathrm{d}^2\bar{v}}{\mathrm{d}t^2}\right|_{t=t_{\mathrm{q}}} > 0\right)$

- When assigning σ_{S} to non-sister cells and σ_{W} to sister cells, the outputted cell-cell contact map is the same as the one generated by the original framework and covers all conserved contacts and non-contacts observed experimentally (Supplementary Fig. 1b).

E. Simulating the 7-cell stage to its quasi-steady state $\left(\left.\frac{\mathrm{d}\bar{v}}{\mathrm{d}t}\right|_{t=t_{\mathrm{q}}} = 0,\ \left.\frac{\mathrm{d}^2\bar{v}}{\mathrm{d}t^2}\right|_{t=t_{\mathrm{q}}} > 0\right)$

- After assigning σ_{S} to non-sister cells and σ_{W} to sister cells, the outputted cell-cell contact map is the same as the one generated by the original framework and covers all conserved contacts and non-contacts observed experimentally (Supplementary Fig. 1c).

F. Simulating the 8-cell stage for a long enough duration (*in silico* time = step number × step size = 15000)

- After assigning σ_{S} to non-sister cells and σ_{W} to sister cells, the simulation with additional relatively weak attraction in ABpl-E contact (i.e., $\sigma_{\mathrm{ABpl,\ E}} = \sigma_{\mathrm{W}}$) generates the typical 8-cell structure stabilized in three-dimensional, whose cell-cell contact map is the same as the one generated by the original framework and covers all conserved contacts and non-contacts observed experimentally (Supplementary Fig. 1d and Supplementary Fig. 3a, b).
- After assigning σ_{S} to non-sister cells and σ_{W} to sister cells, the simulation with defaulted relatively strong attraction in ABpl-E contact (i.e., $\sigma_{\mathrm{ABpl,E}} = \sigma_{\mathrm{S}}$) is unstabilized and collapses into an incorrect structure much earlier than the one with relatively weak attraction (Supplementary Fig. 3a-c).

4. Search result

Given the original spatial resolution $\delta l = 0.25\ \mu\mathrm{m}$ and temporal resolution $\delta t = 0.10$, we repeat the simulation procedure from 1- to 8-cell stages under $\delta l = 0.25\ \mu\mathrm{m}: 0.25\ \mu\mathrm{m}: 2.00\ \mu\mathrm{m}$ and $\delta t = 0.10: 0.05: 0.40$ as shown in Supplementary Fig. 4a. The experimental data of cell division order and axis and volume segregation ratio in *C. elegans* early embryogenesis is obtained from [1] and inputted (Supplementary Table 1). The eggshell boundary is set as a fitted ellipsoid with semi-axes $L_x = 28.28\ \mu\mathrm{m}$, $L_y = 12.87\ \mu\mathrm{m}$, $L_z = 18.81\ \mu\mathrm{m}$ [5], while the region $|y| > w_{\max} = 10.21\ \mu\mathrm{m}$ is removed to resemble the lateral compression in fluorescence imaging; the x, y, and z coordinates of the eggshell center are prescribed as $(30\ \mu\mathrm{m}, 15\ \mu\mathrm{m}, 20\ \mu\mathrm{m})$, and the eggshell structure is shown in Fig. 1a. With the system parameters set according to the values used previously (Supplementary Table 2) [2], the stage when a simulation fails to meet the requirement is shown in Supplementary Fig. 4b, c, revealing the largest degraded resolutions $\delta l = 0.50\ \mu\mathrm{m}$ and $\delta t = 0.30$ that are still at work (Combination ④ in Supplementary Fig. 4b, c). The attraction intensities in the EMS-P2 interface and the others at 4-cell stage are fitted as $\sigma_{\mathrm{W}} = 0$ and $\sigma_{\mathrm{S}} = 0.5$ respectively.

Supplementary Note 2

Iterative process of *MorphoSim*

Given the initial conditions ϕ_i^0, ϕ_i^{n+1} with $n = 0$ (i.e., ϕ_i^1) is computed by the first-order semi-implicit scheme:

$$\frac{\tau}{\delta t}\left(\phi_i^{n+1} - \phi_i^n\right) = \gamma\Delta\phi_i^{n+1} + F_i^n - S\left(\phi_i^{n+1} - \phi_i^n\right), \quad \text{(S1)}$$

where F_i^n is the nonlinear term and expressed by:

$$F_i^n =- \gamma c(4(\phi_i^n)^3 - 6(\phi_i^n)^2 + 2\phi_i^n) - g_{\mathrm{e}}\phi_i^n\phi_{\mathrm{e}}^2 - g\phi_i^n\sum_{j\neq i}^{N}\left(\phi_j^n\right)^2 - \nabla\phi_i^n\cdot\sum_{j\neq i}^{N}\sigma_{i,j}\nabla\phi_j^n + M'\left(1 - \frac{\int_\Omega \phi_i^n \mathrm{d}\boldsymbol{r}}{V_i(t)}\right)|\nabla\phi_i^n| \quad \text{,(S2)}$$

In the simulations of the *synNotch* systems, Gaussian white noise is added to the evolution equation. Thus, the nonlinear term F_i^n turns into:

$$F_i^n =- \gamma c(4(\phi_i^n)^3 - 6(\phi_i^n)^2 + 2\phi_i^n) - g_{\mathrm{e}}\phi_i^n\phi_{\mathrm{e}}^2 - g\phi_i^n\sum_{j\neq i}^{N}\left(\phi_j^n\right)^2 - \nabla\phi_i^n\cdot\sum_{j\neq i}^{N}\sigma_{i,j}\nabla\phi_j^n + M'\left(1 - \frac{\int_\Omega \phi_i^n \mathrm{d}\boldsymbol{r}}{V_i(t)}\right)|\nabla\phi_i^n| - \kappa\sqrt{\delta t}\nabla\phi_i^n \cdot u_i^n \quad \text{,(S3)}$$

where u_i^n is the velocity of the *i*-th cell's random motion sampled from the standard Gaussian distribution at each time step. After ϕ_i^0 and ϕ_i^1 are obtained, ϕ_i^{n+1} with $n \geq 1$ is computed by the second-order semi-implicit scheme:

$$\frac{\tau}{2\delta t}\left(3\phi_i^{n+1} - 4\phi_i^n + \phi_i^{n-1}\right) = \gamma\Delta\phi_i^{n+1} + 2F_i^n - F_i^{n-1} - S\left(\phi_i^{n+1} - 2\phi_i^n + \phi_i^{n-1}\right). \quad \text{(S4)}$$

Supplementary Table 1

Cell Division Group/Order	Dividing Cell	Cell Division Axis (Unit Vector)			Daughter Cell in Vector Head	Daughter Cell in Vector Tail
		Weight in x / A-P Direction	Weight in y / L-R Direction	Weight in z / D-V Direction		
1	P0	-0.999	-0.050	-0.016	AB	P1
2	AB	-0.363	0.606	-0.708	ABa	ABp
3	P1	-0.949	-0.002	-0.316	EMS	P2
4	ABa	-0.377	-0.634	-0.676	ABal	ABar
	ABp	-0.454	-0.873	-0.179	ABpl	ABpr
5	EMS	-0.957	0.271	0.106	MS	E
6	P2	-0.225	-0.094	0.970	C	P3
7	ABal	-0.264	-0.418	0.869	ABala	ABalp
	ABar	-0.497	0.785	-0.369	ABara	ABarp
	ABpl	-0.576	-0.312	0.756	ABpla	ABplp
	ABpr	-0.315	0.021	0.949	ABpra	ABprp
8	MS	-0.941	-0.032	-0.338	MSa	MSp
9	E	-0.927	0.128	-0.353	Ea	Ep
10	C	-0.962	-0.116	-0.249	Ca	Cp
11	ABala	-0.677	0.642	-0.361	ABalaa	ABalap
	ABalp	-0.762	0.584	0.281	ABalpa	ABalpp
	ABara	-0.973	0.084	-0.214	ABaraa	ABarap
	ABarp	-0.969	0.147	0.198	ABarpa	ABarpp
	ABpla	-0.994	-0.009	0.111	ABplaa	ABplap
	ABplp	-0.966	0.164	-0.200	ABplpa	ABplpp
	ABpra	-0.940	0.274	0.203	ABpraa	ABprap
	ABprp	-0.961	0.273	-0.041	ABprpa	ABprpp
12	P3	0.249	-0.142	0.958	D	P4
13	MSa	-0.865	0.413	0.283	MSaa	MSap
	MSp	-0.997	0.010	-0.070	MSpa	MSpp
14	Ca	-0.821	-0.065	0.567	Caa	Cap
	Cp	-0.611	-0.224	0.759	Cpa	Cpp
15	ABalaa	-0.636	-0.309	0.707	ABalaaa	ABalaap
	ABalap	-0.734	-0.332	0.593	ABalapa	ABalapp
	ABalpa	-0.500	-0.462	0.733	ABalpaa	ABalpap
	ABalpp	-0.660	-0.494	0.565	ABalppa	ABalppp
	ABaraa	-0.782	-0.100	0.616	ABaraaa	ABaraap
	ABarap	-0.829	-0.088	0.552	ABarapa	ABarapp
	ABarpa	-0.735	-0.366	0.570	ABarpaa	ABarpap
	ABarpp	-0.903	-0.402	0.150	ABarppa	ABarppp
	ABplaa	-0.623	-0.269	0.734	ABplaaa	ABplaap
	ABplap	-0.664	-0.126	0.737	ABplapa	ABplapp
	ABplpa	-0.809	-0.526	0.261	ABplpaa	ABplpap
	ABplpp	-0.805	-0.492	0.331	ABplppa	ABplppp

	ABpraa	-0.803	-0.398	0.444	ABpraaa	ABpraap
	ABprap	-0.888	-0.221	0.404	ABprapa	ABprapp
	ABprpa	-0.597	-0.040	0.801	ABprpaa	ABprpap
	ABprpp	-0.307	-0.171	0.936	ABprppa	ABprppp
16	Ea	0.113	-0.123	-0.986	Eal	Ear
	Ep	-0.162	-0.460	-0.873	Epl	Epr
17	MSaa	-0.956	-0.121	0.268	MSaaa	MSaap
	MSap	-0.928	-0.360	-0.096	MSapa	MSapp
	MSpa	-0.886	-0.161	0.435	MSpaa	MSpap
	MSpp	-0.772	0.029	0.635	MSppa	MSppp
18	D	-0.251	-0.602	-0.758	Da	Dp
19	Caa	-0.910	-0.082	0.406	Caaa	Caap
	Cap	-0.983	-0.181	0.034	Capa	Capp
	Cpa	-0.643	-0.029	0.765	Cpaa	Cpap
	Cpp	0.386	-0.002	0.923	Cppa	Cppp
20	ABalaaa	-0.248	0.716	-0.652	ABalaaaa	ABalaaap
	ABalaap	-0.958	0.246	-0.149	ABalaapa	ABalaapp
	ABalapa	-0.888	0.428	-0.170	ABalapaa	ABalapap
	ABalapp	-0.976	0.195	0.099	ABalappa	ABalappp
	ABalpaa	-0.732	0.634	0.251	ABalpaaa	ABalpaap
	ABalpap	-0.939	-0.061	0.338	ABalpapa	ABalpapp
	ABalppa	-0.966	-0.050	0.255	ABalppaa	ABalppap
	ABalppp	-0.991	-0.134	0.030	ABalpppa	ABalpppp
	ABaraaa	-0.752	0.408	-0.518	ABaraaaa	ABaraaap
	ABaraap	-0.994	0.085	0.063	ABaraapa	ABaraapp
	ABarapa	-0.998	0.016	0.055	ABarapaa	ABarapap
	ABarapp	-0.887	0.183	0.424	ABarappa	ABarappp
	ABarpaa	-0.842	0.533	0.085	ABarpaaa	ABarpaap
	ABarpap	-0.962	0.248	0.113	ABarpapa	ABarpapp
	ABarppa	-0.873	0.091	0.478	ABarppaa	ABarppap
	ABarppp	-0.863	0.148	0.483	ABarpppa	ABarpppp
	ABplaaa	-0.956	0.157	-0.248	ABplaaaa	ABplaaap
	ABplaap	-0.995	-0.094	0.030	ABplaapa	ABplaapp
	ABplapa	-0.973	-0.173	0.150	ABplapaa	ABplapap
	ABplapp	-0.987	-0.146	0.063	ABplappa	ABplappp
	ABplpaa	-0.950	0.131	-0.282	ABplpaaa	ABplpaap
	ABplpap	-0.962	-0.232	-0.147	ABplpapa	ABplpapp
	ABplppa	-0.980	-0.101	-0.170	ABplppaa	ABplppap
	ABplppp	-0.919	-0.272	-0.285	ABplpppa	ABplpppp
	ABpraaa	-0.857	0.474	0.201	ABpraaaa	ABpraaap
	ABpraap	-0.856	0.461	0.233	ABpraapa	ABpraapp
	ABprapa	-0.876	0.193	0.441	ABprapaa	ABprapap
	ABprapp	-0.490	0.165	0.856	ABprappa	ABprappp
	ABprpaa	-0.852	0.503	0.146	ABprpaaa	ABprpaap

	ABprpap	-0.727	0.133	0.673	ABprpapa	ABprpapp
	ABprppa	-0.727	0.380	0.572	ABprppaa	ABprppap
	ABprppp	-0.017	-0.003	1.000	ABprpppa	ABprpppp
21	MSaaa	-0.985	-0.139	0.106	MSaaaa	MSaaap
	MSaap	-0.993	-0.097	0.065	MSaapa	MSaapp
	MSapa	-0.996	-0.038	0.076	MSapaa	MSapap
	MSapp	-0.993	0.107	0.041	MSappa	MSappp
	MSpaa	-0.934	-0.152	0.323	MSpaaa	MSpaap
	MSpap	-0.952	0.002	0.305	MSpapa	MSpapp
	MSppa	-0.905	-0.088	0.417	MSppaa	MSppap
	MSppp	-0.797	-0.074	0.599	MSpppa	MSpppp
22	P4	0.910	0.090	0.405	Z2	Z3
23	Eal	-0.951	-0.075	0.300	Eala	Ealp
	Ear	-0.946	-0.026	0.324	Eara	Earp
	Epl	-0.928	0.102	0.359	Epla	Eplp
	Epr	-0.865	0.173	0.472	Epra	Eprp
24	Da	-0.964	0.212	-0.163	Daa	Dap
	Dp	-0.624	0.368	0.689	Dpa	Dpp

Note:

1. All the division axes except the ones of P0, AB, and P1 are measured in and averaged over 46 embryo samples from [1].

2. The division axes of P0, AB, and P1 are measured in 1 embryo sample from [6].

3. The order of division groups is estimated using 222 embryo samples from [6].

Cell	Volume (µm^3)
P0'	20768.965
AB'	12304.201
ABa'	6195.266
ABal'	2930.268
ABala'	1300.653
ABalaa'	608.436
ABalaaa'	295.064
ABalaaaa'	118.750
ABp'	6108.935
ABar'	3162.200
ABalp'	1536.119
ABalap'	676.188
ABalaap'	298.768
ABalaaap'	163.790
ABpl'	3155.891
ABara'	1481.336
ABalpa'	786.214
ABalapa'	326.547
ABalaapa'	128.205
ABpr'	2857.238
ABarp'	1639.655
ABalpp'	725.486
ABalapp'	346.528
ABalaapp'	159.158
ABpla'	1815.287
ABaraa'	748.704
ABalpaa'	342.927
ABalapaa'	142.307
ABplp'	1324.941
ABarap'	712.661
ABalpap'	397.841
ABalapap'	164.046
ABpra'	1521.710
ABarpa'	759.423
ABalppa'	304.208
ABalappa'	133.065
ABprp'	1327.680
ABarpp'	842.747
ABalppp'	403.467
ABalappp'	198.751
ABplaa'	925.546
ABaraaa'	329.774

ABalpaaa'	105.056
ABplap'	856.884
ABaraap'	394.227
ABalpaap'	227.551
ABplpa'	697.020
ABarapa'	349.064
ABalpapa'	150.891
ABplpp'	592.583
ABarapp'	331.718
ABalpapp'	229.292
ABpraa'	769.087
ABarpaa'	404.548
ABalppaa'	97.428
ABprap'	707.478
ABarpap'	350.110
ABalppap'	177.389
ABprpa'	680.062
ABarppa'	397.760
ABalpppa'	167.162
ABprpp'	616.146
ABarppp'	421.510
ABalpppp'	229.333
ABplaaa'	379.855
ABaraaaa'	106.927
ABplaap'	515.304
ABaraaap'	201.607
ABplapa'	411.964
ABaraapa'	183.906
ABplapp'	420.797
ABaraapp'	178.995
ABplpaa'	318.943
ABarapaa'	136.151
ABplpap'	344.503
ABarapap'	196.081
ABplppa'	252.349
ABarappa'	146.626
ABplppp'	314.199
ABarappp'	168.724
ABpraaa'	305.353
ABarpaaa'	108.484
ABpraap'	420.393
ABarpaap'	270.377
ABprapa'	340.774
ABarpapa'	159.350

ABprapp'	351.925
ABarpapp'	178.438
ABprpaa'	298.279
ABarppaa'	168.090
ABprpap'	348.439
ABarppap'	207.852
ABprppa'	238.288
ABarpppa'	186.282
ABprppp'	325.126
ABarpppp'	218.135
ABplaaaa'	155.857
ABplaaap'	204.613
ABplaapa'	178.207
ABplaapp'	305.971
ABplapaa'	156.323
ABplapap'	229.918
ABplappa'	244.427
ABplappp'	149.372
ABplpaaa'	140.532
ABplpaap'	148.210
ABplpapa'	132.702
ABplpapp'	196.356
ABplppaa'	103.775
ABplppap'	138.287
ABplpppa'	132.410
ABplpppp'	161.303
ABpraaaa'	119.886
ABpraaap'	173.568
ABpraapa'	139.478
ABpraapp'	264.635
ABprapaa'	115.395
ABprapap'	198.254
ABprappa'	202.398
ABprappp'	129.042
ABprpaaa'	132.854
ABprpaap'	139.799
ABprpapa'	137.607
ABprpapp'	187.918
ABprppaa'	95.931
ABprppap'	139.569
ABprpppa'	134.252
ABprpppp'	168.436
EMS'	4856.202
MS'	2550.275

MSa'	1284.436
MSaa'	738.785
MSaaa'	368.657
MSaaaa'	204.478
MSp'	1306.391
MSap'	516.410
MSaap'	341.298
MSaaap'	174.321
MSpa'	782.767
MSapa'	255.031
MSaapa'	210.321
MSpp'	485.832
MSapp'	229.688
MSaapp'	115.168
MSpaa'	363.034
MSapaa'	115.580
MSpap'	383.363
MSapap'	149.893
MSppa'	251.647
MSappa'	98.052
MSppp'	222.945
MSappp'	130.363
MSpaaa'	236.716
MSpaap'	121.860
MSpapa'	225.435
MSpapp'	142.187
MSppaa'	109.978
MSppap'	141.764
MSpppa'	94.841
MSpppp'	116.543
E'	2248.888
Ea'	1157.802
Eal'	568.408
Eala'	303.612
Ep'	1070.608
Ear'	557.113
Ealp'	262.558
Epl'	533.749
Eara'	293.750
Epr'	532.455
Earp'	267.991
Epla'	268.353
Eplp'	270.884
Epra'	258.213

Eprp'	271.820
C'	2368.228
Ca'	1093.079
Caa'	587.487
Caaa'	419.151
Cp'	1266.189
Cap'	474.180
Caap'	135.246
Cpa'	756.062
Capa'	217.713
Cpp'	469.913
Capp'	214.333
Cpaa'	434.240
Cpap'	292.509
Cppa'	214.793
Cppp'	221.036
D'	779.917
Da'	367.618
Daa'	155.095
Dp'	383.632
Dap'	224.557
Dpa'	156.075
Dpp'	230.331
P1'	8464.764
P2'	3608.562
P3'	1157.047
P4'	337.725
Z2'	152.924
Z3'	154.799

Note:

1. All the volumes except the ones of P0, AB, and P1 are measured in and averaged over 17 embryo samples from [1].

2. The volume of AB is calculated with the sum of the volumes of ABa and ABp.

3. The volume of P1 is calculated with the sum of the volumes of EMS and P2.

4. The volume of P0 is calculated with the sum of the volumes of AB and P1.

Supplementary Table 2

Significance	Mathematical Symbol	Value Assignment
Number of Grid Node	Ω	Variable for Scanning
Spatial Grid Size/Spatial Resolution	δl	Variable for Scanning
Time Step Size/Temporal Resolution	δt	Variable for Scanning
Relatively Strong Cell-Cell Attraction	σ_{S}	Fitted by Cell-Cell Contact Areas at 4-Cell Stage
Relatively Weak Cell-Cell Attraction	σ_{W}	Fitted by Cell-Cell Contact Areas at 4-Cell Stage
Cell Surface Tension	γ	0.25
Cell-Eggshell Repulsion	g_{e}	16
Cell-Cell Repulsion	g	1.6
Cell Boundary Thickness	c	1
Volume Constriction Strength	M	0.0012
Ambient Viscosity	τ	2.62
Width of Initial Interface between Sister Cells	ε	2^{-52}

Note:

1. The value assignment for the parameters are adopted from [2].

2. The scanning method for spatial grid size and time step size is introduced in Supplementary Note 1 and Supplementary Fig. 4a-c.

3. The fitting method for strong/weak cell-cell attraction is introduced in Supplementary Note 1 and Supplementary Fig. 2a, b.

Supplementary Table 3

1st Order		Computing Time (s)			
δt	*S*	1- to 4-Cell Stage	6-Cell Stage	7-Cell Stage	8-Cell Stage
0.3	0.0	629.134	63.525	47.666	896.890
0.4	1.4	524.092	57.495	43.371	817.180
0.5	2.7	470.385	56.693	43.437	810.301
0.6	3.6	428.590	56.027	44.135	808.479
0.7	4.2	391.190	54.995	44.367	804.458
0.8	4.6	358.324	53.911	44.353	789.881
0.9	5.0	330.259	53.478	45.294	794.167
1.0	5.3	303.752	52.859	46.221	813.019
1.1	5.5	282.537	53.693	48.320	813.888
1.2	5.7	249.279	53.437	49.995	816.188
1.3	5.9	228.060	52.975	51.726	822.181
1.4	6.1	216.145	52.766	54.245	833.777
1.5	6.2	204.913	51.919	56.225	838.283
1.6	6.3	195.931	51.391	59.371	849.788
1.7	6.4	187.856	50.809	65.445	881.881
1.8	6.5	180.639	50.114	79.770	950.127
1.9	6.5	177.826	49.627	90.385	1000.491
2.0	6.6	165.613	49.237	66.934	868.794

2nd Order		Computing Time (s)			
δt	***S***	**1- to 4-Cell Stage**	**6-Cell Stage**	**7-Cell Stage**	**8-Cell Stage**
0.3	4.0	631.262	64.792	47.910	992.210
0.4	6.1	473.535	48.069	35.923	744.964
0.5	7.4	387.690	39.949	29.233	612.930
0.6	8.3	317.401	32.433	24.057	497.552
0.7	8.9	271.550	27.955	20.659	426.129
0.8	9.4	238.140	24.617	18.160	373.920
0.9	9.8	212.236	21.913	16.187	331.689
1.0	10.0	193.727	20.153	14.810	305.900
1.1	10.3	173.246	17.938	13.355	270.361
1.2	10.5	159.699	16.574	12.148	250.908
1.3	10.7	146.310	15.299	11.249	230.295
1.4	10.8	135.235	14.056	10.493	213.225
1.5	10.9	128.929	13.460	9.940	203.109
1.6	11.0	119.002	12.464	9.165	185.137
1.7	11.1	116.914	11.817	8.736	176.195
1.8	11.2	106.368	10.983	8.111	165.382
1.9	11.3	100.805	10.560	7.727	157.116
2.0	11.4	97.098	10.279	7.535	153.339
2.1	11.4	90.899	0.299	0.381	143.138
2.2	11.4	87.321	0.303	0.344	137.496
2.3	11.5	82.637	0.325	0.372	128.968
2.4	11.5	79.196	0.302	0.382	125.525
2.5	11.6	78.006	0.334	0.385	123.465

Baseline		Computing Time (s)			
δt	***S***	**1- to 4-Cell Stage**	**6-Cell Stage**	**7-Cell Stage**	**8-Cell Stage**
0.3	/	629.134	63.525	47.666	896.890

Supplementary Table 4

2nd Order		*in silico* Time $n_t\delta t$			
δt	S	1- to 4-Cell Stage	6-Cell Stage	7-Cell Stage	8-Cell Stage
0.3	0.0	25225	1426	901	59895
0.4	1.4	28180	1730	1110	72170
0.5	2.7	31590	2130	1390	88680
0.6	3.6	34406	2525	1690	105178
0.7	4.2	36583	2901	1989	120873
0.8	4.6	38160	3254	2275	135715
0.9	5.0	39353	3623	2614	151381
1.0	5.3	39860	3980	2960	166350
1.1	5.5	39442	4307	3297	179883
1.2	5.7	37008	4646	3696	192864
1.3	5.9	35899	4996	4150	201456
1.4	6.1	36544	5351	4684	216462
1.5	6.2	37215	5652	5220	229995
1.6	6.3	37901	5962	5914	244090
1.7	6.4	38548	6273	6894	258791
1.8	6.5	39159	6588	8892	274518
1.9	6.5	41857	6840	10612	73530
2.0	6.6	40100	7150	8310	74010

Supplementary Table 5

Parameters	Spatial Scale *L*	
	Original: 64 (μm)	Rescaled: 256 (μm)
c	2	0.125
γ	0.25	4
M'	8	32
g_e	16	16
g	1.6	1.6
	0.9	14.4
σ	0.5	8
	0.3	4.8
τ	2.62	2.62
M'	8	32
κ	0.224	0.894

Supplementary Table 6

$\eta_{AB} = S_{AB}^{1/2} / V_{AB}^{1/3}$		γ_{AB}									
		0.1	0.2	0.3	0.4	0.5	0.6	0.7	0.8	0.9	1.0
γ_{P1}	0.1	2.296	2.279	2.270	2.265	2.261	2.258	2.256	2.254	2.252	2.250
	0.2	2.314	2.292	2.281	2.274	2.268	2.265	2.261	2.259	2.256	2.254
	0.3	2.326	2.301	2.288	2.280	2.274	2.269	2.266	2.263	2.260	2.258
	0.4	2.336	2.308	2.294	2.285	2.279	2.273	2.269	2.266	2.263	2.261
	0.5	2.343	2.314	2.299	2.289	2.282	2.277	2.273	2.269	2.266	2.263
	0.6	2.349	2.319	2.304	2.293	2.286	2.280	2.275	2.271	2.268	2.265
	0.7	2.354	2.324	2.307	2.297	2.289	2.283	2.278	2.274	2.270	2.267
	0.8	2.359	2.327	2.310	2.299	2.291	2.285	2.280	2.276	2.272	2.269
	0.9	2.362	2.331	2.313	2.302	2.294	2.287	2.282	2.277	2.274	2.270
	1.0	2.366	2.333	2.316	2.304	2.296	2.289	2.284	2.279	2.275	2.272

$\eta_{P1} = S_{P1}^{1/2} / V_{P1}^{1/3}$		γ_{AB}									
		0.1	0.2	0.3	0.4	0.5	0.6	0.7	0.8	0.9	1.0
	0.1	2.278	2.303	2.320	2.333	2.344	2.352	2.359	2.365	2.371	2.375
	0.2	2.255	2.273	2.286	2.296	2.304	2.311	2.317	2.322	2.326	2.330
	0.3	2.244	2.258	2.269	2.277	2.284	2.290	2.295	2.299	2.303	2.307
	0.4	2.237	2.249	2.258	2.265	2.271	2.276	2.281	2.285	2.288	2.291
	0.5	2.233	2.243	2.250	2.257	2.262	2.267	2.271	2.274	2.277	2.280
γ_{P1}	0.6	2.229	2.238	2.245	2.250	2.255	2.259	2.263	2.266	2.269	2.271
	0.7	2.226	2.234	2.240	2.245	2.250	2.253	2.257	2.260	2.262	2.265
	0.8	2.224	2.231	2.237	2.241	2.245	2.248	2.252	2.254	2.257	2.259
	0.9	2.222	2.228	2.233	2.238	2.241	2.244	2.247	2.250	2.252	2.254
	1.0	2.220	2.226	2.231	2.235	2.238	2.241	2.244	2.246	2.248	2.250

Legends of Supplementary Tables

Supplementary Table 1. The experimentally-measured cell division order and axis and cell volume in 1- to 102-cell *C. elegans* embryogenesis.

Supplementary Table 2. The system parameter and value assignment in the phase field models with scanned spatial and temporal resolutions.

Supplementary Table 3. The computing time for 1- to 8-cell stages in the phase field models with the first- and second-order schemes.

Supplementary Table 4. The *in silico* time for 1- to 8-cell stages in the phase field models with the first-order scheme.

Supplementary Table 5. The parameter setting for adjusting the spatial scale of *synNotch* simulations to approximate the experimental ones.

Supplementary Table 6. The irregularity score of AB and P1 cells at 2-cell stage with their γ values being varied.

Legends of Supplementary Movies

Supplementary Movie 1. Phase-field simulation of *C. elegans* embryogenesis from 12- to 24-cell stages, before modifying the formulation of cell volume constriction. The cell disappearance occurs in the P4 cell at 24-cell stage, as shown in Fig. 3a.

Supplementary Movie 2. Phase-field simulation of *C. elegans* embryogenesis from 1- to 102-cell stages, after modifying the formulation for cell volume constriction. No cell disappearance occurs throughout the simulation, as shown in Fig. 5b.

Supplementary Movie 3. Phase-field simulation of the *synNotch* system with spherically asymmetric separation (two cell types), corresponding to the 2nd row in Fig. 6. The cell types and corresponding colors and value assignments on adhesion are listed in Table 2. The simulation lasts for an *in silico* time = 0 ~ 50000.

Supplementary Movie 4. Phase-field simulation of the *synNotch* system with spherically asymmetric separation (three cell types), corresponding to the 1st row in Fig. 6. The cell types and corresponding colors and value assignments on adhesion are listed in Table 2. The simulation lasts for an *in silico* time = 0 ~ 50000; since *in silico* time = 40000, the red cells which contact at least one green cell are painted blue.

Supplementary Movie 5. Phase-field simulation of the *synNotch* system with spherically asymmetric separation (two cell types), corresponding to the 3rd row in Fig. 6. The cell types and corresponding colors and value assignments on adhesion are listed in Table 2. The simulation lasts for an *in silico* time = 0 ~ 50000.

Supplementary Movie 6. Phase-field simulation of the *synNotch* system with self-repairing, corresponding to the 4th row in Fig. 6. The cell types and corresponding colors and value assignments on adhesion are listed in Table 2. The initial state is adopted from the final state in the simulation of spherically asymmetric separation (two cell types) (3rd row in Fig. 6; Supplementary Movie 5), where the cells with $z < 0$ or without contact to the cell aggregate are removed. The simulation lasts for an *in silico* time = 0 ~ 50000.

Supplementary Movie 7. Phase-field simulation of the *synNotch* system with dissociation, corresponding to the 5th row in Fig. 6. The cell types and corresponding colors and value assignments on adhesion are listed in Table 2. The initial state is adopted from the final state in the simulation of spherically asymmetric separation (two cell types) (3rd row in Fig. 6; Supplementary Movie 5). The simulation lasts for an *in silico* time = 0 ~ 50000.

Supplementary References

[1] Cao, J., et al. Establishment of a morphological atlas of the *Caenorhabditis elegans* embryo using deep-learning-based 4D segmentation. *Nat. Commun.* **11**, 6254 (2020).

[2] Kuang, X., et al. Computable early *Caenorhabditis elegans* embryo with a phase field model. *PLoS Comput. Biol.* **18**, e1009755 (2022).

[3] Yamamoto, K. & Kimura, A. An asymmetric attraction model for the diversity and robustness of cell arrangement in nematodes. *Development* **144**, 4437-4449 (2017).

[4] Dutta, P., Odedra, D. & Pohl, C. Planar asymmetries in the *C. elegans* embryo emerge by differential retention of aPARs at cell-cell contacts. *Front. Cell Dev. Biol.* **7**, 209 (2019).

[5] Moshtagh, N. Regular Polygons. File at https://www.mathworks.com/matlabcentral/fileexchange/16608-regular-polygons (2022).

[6] Guan, G., et al. System-level quantification and phenotyping of early embryonic morphogenesis of *Caenorhabditis elegans*. Preprint at https://doi.org/10.1101/776062 (2019).
